# Single-cell transcriptomics of vascularized human brain organoids decipher lineage-specific stress adaptation in fetal hypoxia-reoxygenation injury

**DOI:** 10.7150/thno.117001

**Published:** 2025-06-09

**Authors:** Simeng Yi, Min Huang, Chunmei Xian, Xi Kong, Shigang Yin, Jianhua Peng, Yong Zhang, Xiuju Du, Yong Jiang, Bingqing Xie, Huangfan Xie

**Affiliations:** 1 Laboratory of Neurological Diseases and Brain Function, The Affiliated Hospital, Southwest Medical University, Luzhou, 646000, China.; 2 Institute of Epigenetics and Brain Science, Southwest Medical University, Luzhou, 646000, China.; 3 Department of Neurosurgery, The Affiliated Hospital, Southwest Medical University, NO. 25 of Taiping Street, Luzhou, Sichuan, 646000, China.; 4 Sichuan Provincial Women's and Children's Hospital / The Affiliated Women's and Children's Hospital of Chengdu Medical College, Chengdu, 610041, China.; 5 Research Center for Healthcare Data Science, Zhejiang Lab, Hangzhou, 310000, China.

**Keywords:** fetal hypoxia, vascularized brain organoid, blood-brain barrier, single-cell RNA-sequencing, GABAergic neuron

## Abstract

**Rationale:** Fetal hypoxia, a major contributor to neonatal mortality, induces complex neurovascular disruptions in developing brains, yet human-specific cellular mechanisms remain poorly understood due to limitations in existing models. This study establishes an advanced vascularized human cortical organoid (vhCO) model to decode cell type-specific injury mechanisms and therapeutic targets during hypoxia-reoxygenation.

**Methods:** We developed vhCOs by integrating cortical and vascular organoids, recapitulating mid-to-late gestational neurodevelopment with diverse lineages—neural progenitors, neurons, microglia, and functional vasculature with blood-brain barrier properties. Hypoxia-reoxygenation experiments were conducted on vhCOs, followed by single-cell transcriptomic profiling to dissect cellular responses.

**Results:** Key findings include: (1) Lineage-specific vulnerabilities: astrocyte precursors exhibited developmental arrest, while immature GABAergic neurons (Subtype I) underwent neurogenic collapse. Microglia displayed a biphasic inflammatory response—initially suppressed, then hyperactivated post-reoxygenation, diverging from animal models; (2) Hypoxia memory persisted in non-neural cells (pericytes, fibroblasts), driving compartment-specific vascular remodeling via Notch signaling and collagen deposition; (3) Rewired neural-non-neural crosstalk networks (e.g., IGF2-IGF2R, LGALS3-MERTK, Wnts-SFRP2) revealed novel repair targets inaccessible to conventional models.

**Conclusions:** By prioritizing single-cell resolution, this study delineates human-specific neurovascular pathophysiology and stress adaptation networks in hypoxic brain injury. The vhCO platform bridges translational gaps, offering a paradigm for precision therapeutics and advancing research on developmental brain disorders.

## Introduction

Fetal hypoxia during pregnancy is a common adverse condition in humans that can have lasting effects on multiple physiological systems, including the brain 1, 2. Specifically, the fetal brain, due to its developmental immaturity, is highly susceptible to hypoxic injury, resulting in over 23% of affected newborns experiencing permanent brain damage 3. Research has increasingly shown that fetal hypoxia can impair brain development and lead to a range of behavioral disorders later in life, such as cognitive impairment, depression, anxiety, and other neurological conditions 4-6. There is a critical need to understand the mechanisms of hypoxic brain injury to develop effective treatments. Animal models have been used to study hypoxic brain injury, but they have limitations, including behavioral variability and differences between species 1, 7.

In recent years, three-dimensional (3D) brain organoids derived from human pluripotent stem cells (hPSCs) have emerged as a new robust *in vitro* platform to model brain disorders 8, 9. These organoids replicate key features of the developing human brain and allow for direct observation of processes that cannot be seen *in vivo* 10. Recent studies leveraging these brain organoids have successfully simulated hypoxic conditions, uncovering hypoxia-induced perturbations in cortical cell division, fate specification, and marker expression 11-13. However, these models suffer from two critical limitations: (1) They lack vascular systems and immune components (e.g., microglia), compromising their ability to recapitulate neurovascular interactions and inflammation—key processes in hypoxic brain injury 14; (2) Reliance on bulk RNA-seq and immunostaining obscures cell-type-specific responses, failing to resolve transcriptional heterogeneity across neural and non-neural lineages. These gaps hinder mechanistic insights into how distinct cellular subpopulations adapt to or succumb to hypoxia-reoxygenation stress.

In this study, we developed vascularized human cortical organoids (vhCOs) by integrating hPSC-derived cortical organoids (hCOs) with blood vessel organoids (hBVOs). These vhCOs recapitulated transcriptional profiles of early-to-mid gestation fetal brain cells, including neural progenitors, neurons, microglia, and vascular components with blood-brain-barrier (BBB)-like structure. When exposed to hypoxia-reoxygenation (48 h at 1% O₂ followed by 5 days of normoxia), single-cell transcriptomic analysis revealed cell-type-specific responses within vhCOs: astrocyte precursors (AstPs) and GABAergic neurons subtype I exhibited developmental arrest and functional impairment; microglia showed biphasic inflammation linked to delayed neuroinflammation; non-neural lineages sustained hypoxia signaling while coordinating compartmentalized vascular remodeling; *CellPhoneDB* and SCENIC analyses uncovered hypoxia-reoxygenation-driven rewiring of ligand-receptor interactions and transcription factor (TF) networks that regulates stress adaptation and lineage-specific functional shifts. These findings establish vhCOs as a physiologically robust model for fetal hypoxia-reoxygenation injury, identifying vulnerable cell populations and lineage-specific intricate repair mechanisms, providing mechanistic insights for targeting neurovascular dysfunction in fetal brain hypoxia-reoxygenation injury.

## Materials and Methods

### Key resources

Detailed information on reagents, consumables, software, and algorithms used in this study is provided in Table S1.

### Human embryonic stem cell (hESC) culture

Unmodified H9 hESC line (purchased from Wicell) and *AAVS1-EGFP* H9 cells (with the *EGFP* cassette integrated into the *AAVS1* safe harbor locus for stable EGFP labeling) were cultured on Matrigel-coated dishes (Corning, 354230) in Pluripotency Growth Master 1 (PGM1; Cellapybio, CA1007500) medium supplemented with 1% penicillin-streptomycin (10,000 U/mL; Gibco, 15140-122) at 37 °C/5% CO₂. Medium was replaced daily, and cells were passaged every 5 days using 0.5 mM EDTA (Sigma, 60004). Mycoplasma contamination was routinely monitored.

### Human cortical organoid (hCO) induction and culture

Unmodified H9 cells were dissociated with Accutase, resuspended in PGM1 medium containing 10 μM Y27632, and seeded into Elplasia plates (7,500 cells/aggregate in 200 μL/well) to generate embryoid bodies (EBs). On day 0, EBs were transferred to 6-well ultra-low attachment plates containing ectodermal induction medium (Essential 6 supplemented with 2.5 μM dorsomorphin, 10 μM SB-431542, and 2.5 μM XAV-939) at <40 EBs/well to minimize fusion. During EB culture, gentle manual shaking was performed to ensure that cell aggregates were evenly distributed in the culture medium, thereby preventing fusion via cell-cell contact. From days 2-6, medium was replaced daily. On day 6, cultures were transitioned to neural induction medium (Neurobasal A, 2% B-27 [without vitamin A], 1% alanyl-glutamine, 20 ng/mL epidermal growth factor [EGF], 20 ng/mL basic fibroblast growth factor [bFGF]), refreshed daily until day 16 and every other day until day 25. From day 25, differentiation medium (Neurobasal A, 2% B-27 [without vitamin A], 1% alanyl-glutamine, 20 ng/mL brain-derived neurotrophic factor [BDNF], 20 ng/mL NT3) was replenished every 48 h until day 43. Terminal maturation commenced on day 43 using growth factor-free neural medium (Neurobasal A, 2% B-27 [-vitamin A], 1% alanyl-glutamine), replaced every 3-4 days.

### Human blood vessel organoid (hBVO) induction and culture

Unmodified or *AAVS1-EGFP* H9 cells were dissociated with Accutase, resuspended in PGM1 medium containing 10 μM Y27632, and seeded into Elplasia plates (7,000 cells/aggregate in 200 μL/well) to generate EBs. On the following day (designated day 0), the medium was replaced with mesodermal induction medium, consisting of RPMI 1640 basal medium containing 1% alanyl-glutamine, 1% non-essential amino acids (NEAA), 1% B-27 supplement (without vitamin A), 0.06% (v/v) polyvinyl alcohol (PVA), 50 nM ascorbic acid-2-phosphate, and 6 μM CHIR99021. The mesodermal induction phase was maintained from day 0 to day 2, after which, on day 2, endothelial induction was initiated by replacing CHIR99021 with 50 ng/mL vascular endothelial growth factor (VEGF), 25 ng/mL bone morphogenetic protein 4 (BMP4), and 10 ng/mL bFGF, which were maintained through day 5. From day 5 to day 10, EBs were cultured in MV2 medium with 50 ng/mL VEGF, refreshed every 48 h. At day 10, EBs were embedded in Matrigel droplets and cultured in MV2 basal medium supplemented with 1% B-27 (without vitamin A), 1 × N2 supplement, and 50 ng/mL VEGF. Notably, from day 0 onward, organoids were transferred to 6-well ultra-low attachment plate (with fewer than 40 aggregates per well) to prevent fusion, and all suspension cultures were maintained under normoxic conditions (5% CO₂, 37 °C).

### Vascularized human cortical organoids (vhCOs) preparation and culture

To generate vhCOs, two hBVOs at day 10 and one hCO at day 30 were collected and embedded together in a single 30 μL droplet of Matrigel. The two hBVOs were positioned on opposite sides of the hCO, with pipette tips used to adjust their configuration. The droplet containing the organoid assembly was then transferred to a 24-well ultra-low attachment plate for further cultivation in co-culture medium, which consisted of a 1:1 mixture of MV2 complete medium and Neurobasal A medium, supplemented with 1% B-27 (without vitamin A), 1% alanyl-glutamine, 5% fetal bovine serum, 50 ng/mL VEGF, and 10 ng/mL bFGF. After allowing the organoid assembly to maintain close contact for 3 days to ensure stabilization without dissociation, the Matrigel droplet containing the consolidated organoid complex was transferred to a 24-well ultra-low attachment plate. The co-culture system was maintained in a specialized medium comprising a 1:1 mixture of MV2 complete medium and Neurobasal-A medium that was supplemented with 1% B-27 supplement (without vitamin A), 1% alanyl-glutamine, 5% fetal bovine serum, 50 ng/mL VEGF, and 10 ng/mL bFGF. Medium renewal was performed every 2-3 days. The cultures were incubated at 37 °C in a humidified atmosphere containing 5% CO_2_ for 30 days, with strict adherence to aseptic techniques throughout the cultivation period. Successful fusion was confirmed in all organoids used for downstream assays via GFP tracking of vascular networks (hBVO-derived) and bright-field morphological integration. Total vessel length per organoid was quantified from one fluorescence microscopic field. GFP-labeled vasculature was analyzed using AngioTool (version 0.6a [64 bits], October 2014; National Cancer Institute), a software which measures cumulative vessel length in pixels. This value was converted to micrometers using the formula: Length (μm) = Total pixels / 2.842129, where 2.842129 pixels/μm represents our microscope-calibrated spatial resolution.

### Modeling hypoxia-reoxygenation injury using vhCOs

To model hypoxia-reoxygenation injury, vhCOs at day 60 (30 days post-fusion with hBVOs) were transferred from normoxic conditions (21% O₂, 5% CO₂) to a hypoxia incubator (ESCO, CCL-170T-8; 1% O₂, 5% CO₂, 37 °C) for 48 h using co-culture medium that was pre-equilibrated approximately 16 h at 1% O₂, 5% CO₂, and 37 °C. vhCOs were then either immediately collected for post-hypoxia analysis or returned to normoxia (21% O₂, 5% CO₂, 37 °C) for 5 days to assess reoxygenation effects, where all samples were collected, fixed or processed for downstream analyses.

### Cryogenic tissue processing

The collected organoid samples were fixed in 4% paraformaldehyde (PFA) at 4 °C overnight, then washed three times with phosphate-buffered saline (PBS) and dehydrated in 30% sucrose at 4 °C for 24-48 h. Subsequently, hCOs were embedded in a 1:1 mixture of optimal cutting temperature (OCT) compound and 30% sucrose, and cryosectioned into 12-16 μm slices. For hBVOs and vhCOs, samples were incubated in a preheated gelatin solution comprising Dulbecco's phosphate-buffered saline (DPBS) with 10% sucrose and 7.5% gelatin at 37 °C for 1 h, allowing the gelatin to permeate the Matrigel droplets and completely envelop the organoids. Following incubation, the organoids were rapidly transferred into embedding molds and snap-frozen. The optimal sectioning temperature for gelatin-embedded organoids was maintained between -26 °C and -30 °C, and section thickness was adjusted according to imaging requirements, with slices ranging from 20 to 30 μm produced using a cryostat (Leica, CM3050-S).

### Immunofluorescence

Cryosections were dried at room temperature, then subjected to antigen retrieval by boiling in a citrate-based buffer for 10 min, followed by a cooling period of at least 20 min. Sections were subsequently washed three times with PBS and incubated with a blocking solution containing 10% normal donkey serum, 0.25% Triton X-100, and 1% bovine serum albumin. Primary antibodies were diluted 1:100-1:200 in an antibody buffer (DPBS supplemented with 3% normal donkey serum, 1% bovine serum albumin, and 0.1% Triton X-100) and applied overnight at 4 °C. The next day, sections were washed three times with PBS containing 0.025% Tween-20, and incubated with secondary antibodies conjugated to Alexa Fluor 488, 594, or 647 (diluted 1:500) and DAPI nuclear stain (1:1,000) in antibody buffer for 2 h at room temperature. After incubation, sections were mounted with an appropriate mounting medium and stored at 4 °C prior to imaging.

For whole-mount staining, organoids were fixed in 4% PFA at 4 °C overnight, washed three times with PBS, and incubated in a blocking solution at room temperature for 2 h. Primary anti-human antibodies were then added to the antibody buffer and incubated with the samples at 4 °C for over 48 h. After three sequential 20-min PBS-T washes (PBS with 0.05% Tween-20), the samples were further incubated with appropriate secondary antibodies at 4 °C for an additional 48 h. Nuclear counterstaining was performed using Hoechst 33342 for 15 min at room temperature. Following final PBS washes (3×10 min), the stained organoids were subjected to confocal imaging. Z-stack images acquired through the organoids were merged to generate two-dimensional maximum intensity projections using ZEN blue imaging software. All imaging parameters (laser power, gain, pinhole size) were maintained constant across comparative samples.

### RNA isolation and reverse transcription quantitative PCR (RT-qPCR)

Total RNA was extracted from hCO or vhCO pools (n≥3 per experimental group) using TRIzol™ reagent. Complementary DNA (cDNA) synthesis was carried out using 2 × SuperMix Reverse Transcriptase, following the manufacturer's recommended protocol. RT-qPCR was performed with SYBR™ Green Master Mix on a qTower³G real-time PCR system (Analytik Jena). The amplification protocol comprised an initial denaturation at 95 °C for 5 min, followed by 40 cycles of 95 °C for 15 s, and annealing/extension at 60 °C for 20 s. Gene expression data were normalized to endogenous reference genes (specified in respective figure legends) and analyzed using the comparative 2-ΔΔCT method relative to normoxic controls. Primer sequences for the RT-qPCR are listed in Table S1.

### Single-cell RNA sequencing (scRNA-seq)

Single-cell suspensions were prepared from hypoxic, reoxygenated, or normoxic vhCOs by enzymatic dissociation with papain, achieving a concentration of 2 × 10⁵ cells per milliliter in ice-cold D-PBS. These cells were then processed using the GEXSCOPE Single-Cell RNA Library Kit Tissue from Singleron Biotechnologies (Nanjing, China) to barcode individual cells, capture their mRNA, and create cDNA libraries. These libraries were diluted to 4 ng/mL, combined, and sequenced on an Illumina NovaSeq 6000 platform (San Diego, CA, USA) using 150-base pair paired-end reads for subsequent scRNA-seq analysis.

### scRNA-seq data analysis

In the analysis of scRNA-seq data, the following steps were undertaken:

(1) Data Preprocessing and Quality Control: Single-cell transcriptome data were read in and analyzed using the R Seurat package (v4.4.0). Genes expressed in at least 3 cells (min.cells=3) and cells with at least 200 detected genes (min.features=200) were retained to construct the Seurat object. Quality control was performed by calculating the percentage of mitochondrial gene expression (genes starting with "MT-"), and cells with high mitochondrial content (>=30%) were excluded. The NormalizeData function was used for log-normalization (scale factor=10,000).

(2) Uniform Manifold Approximation and Projection (UMAP) Cell Clustering: The gene expression matrix was scaled (ScaleData), and principal component analysis (PCA) was performed to obtain feature vectors. A K-nearest neighbor graph (k=20) was constructed based on the PCA-reduced dimensions, and cell clustering was performed using the Louvain algorithm (resolution=1.2). The spatial distribution of cell subpopulations was visualized using the UMAP algorithm. Differentially expressed genes (DEGs) were analyzed using the Wilcoxon rank-sum test (requiring at least 25% of cells expressing the gene, i.e., min.pct=0.25, and log2FC.threshold=0.25).

(3) Visualization and Functional Analysis: Key results were presented using the DimPlot function (for UMAP visualization), VlnPlot function (for violin plots of gene expression), and Dotplot function (for dot plots of gene expression) in the Seurat R package. Gene Ontology (GO) and Kyoto Encyclopedia of Genes and Genomes (KEGG) pathway enrichment analyses were performed using the clusterProfiler package (v4.10.1).

(4) Gene Set Enrichment Analysis (GSEA): For pseudo-bulk GSEA, average gene expression values per condition were calculated using the AverageExpression function in the Seurat R package. Genes were ranked by fold change (FC) between conditions to generate a ranked list, which was analyzed for coordinated pathway changes using predefined Hallmark gene sets from the MSigDB database. This analysis utilized the GSEA and gseKEGG functions within the clusterProfiler R package (v4.10.1) to assess Hallmark and KEGG pathways, respectively. For cell type-specific GSEA, differential expression analysis of cell subpopulations was performed using the Wilcoxon rank-sum test, with log2 fold change (log2FC) as the ranking metric. Predefined Hallmark, GO, and KEGG gene sets were similarly analyzed using the GSEA, gseGO, and gseKEGG functions in clusterProfiler. Venn diagrams were generated using the eulerr R package (v7.0.2).

(5) STREAM Pseudotime Analysis: STREAM (Single-cell Trajectories Reconstruction, Exploration and Mapping, v1.1) was used for pseudotime analysis. First, UMI counts were normalized based on library size, and mitochondrial genes were filtered out before dimensionality reduction. Then, an improved locally linear embedding method was used to embed the top 2,000 highly variable genes. The number of neighboring cells used in the analysis was set to 100. This process generated a two-dimensional tree and pathway mapping for visualizing the mapped cell lineages.

(6) Cell-Cell Communication Analysis: Ligand-receptor interaction-mediated cell-cell communication was analyzed using CellPhoneDB (v4.0.0) with default parameters and 1,000 iterations. The top 10 condition-specific ligand-receptor interactions were visualized in bubble plots, where dot size corresponds to interaction scores calculated by *CellPhoneDB*. Cell-cell interaction networks were visualized using the netVisual_circle function in the CellChat R package, with line thickness representing the number of ligand-receptor pairs.

(7) Monocle2 Pseudotime Analysis: Analysis was performed using the monocle (v2.30.1) R package 15. Genes with an average expression level ≥0.5 were retained. The top 400 genes with corrected *p*-values were selected as highly variable genes for trajectory inference. The DDRTree (Discriminative Dimensionality Reduction with Trees) algorithm was used for nonlinear dimensionality reduction, and a minimum spanning tree (MST) model was constructed to capture the topological structure of cell states. Pseudotime values were calculated using the orderCells function, with the cell closest to the root node serving as the developmental starting point. The plot_cell_trajectory function was used to draw a 2D trajectory plot colored by pseudotime or cell subpopulation. The plot_genes_branched_heatmap was used to display the dynamic expression patterns of the top 100 genes with adjusted *p*-values.

(8) Cell State-Specific Gene Regulatory Network Analysis: Analysis was performed using pySCENIC (v0.12.1). The GRNBoost2 algorithm was used to infer co-expression relationships between genes to construct a co-expression network, with a threshold set to retain interactions with a weight ≥ 0.001. The ctx-clustering algorithm was used to identify co-expression modules (number of modules = 50). The AUCell algorithm was used to quantify regulatory module activity and calculate cell activity state scores. The SCENIC R package (v1.3.1) was used for visualization of the results in bubble plots, scatter plots, and heatmaps. The TF regulatory network was visualized using Cytoscape (v3.9.0). For each cell subpopulation, the log2FoldChange was calculated for hypoxia_48h.vs. normoxia_48h and hypoxia_7d.vs. normoxia_7d, and genes with |log2FoldChange|>0.585 (FoldChange>1.5) were selected as candidate regulatory genes for TFs. If a TF had more than 50 candidate regulatory genes, the top 50 genes with the highest log2FoldChange were selected for network visualization. In the network, the color of the gene nodes represents upregulation (red) and downregulation (blue).

### Quantification and statistics analysis

Statistical analyses were performed using GraphPad Prism 10.0 or Microsoft Excel 2019. Data were analyzed using two-tailed Student's *t*-tests or one-way ANOVA, with *P* < 0.05 considered statistically significant. Sample sizes (*n*) are detailed in figure legends. Bar and line graphs represent mean ± SD unless noted. Immunofluorescence images are representative of at least two independent experimental replicates under specified conditions.

## Results

### Establishment of vhCOs from hESCs

**Figure 1:**
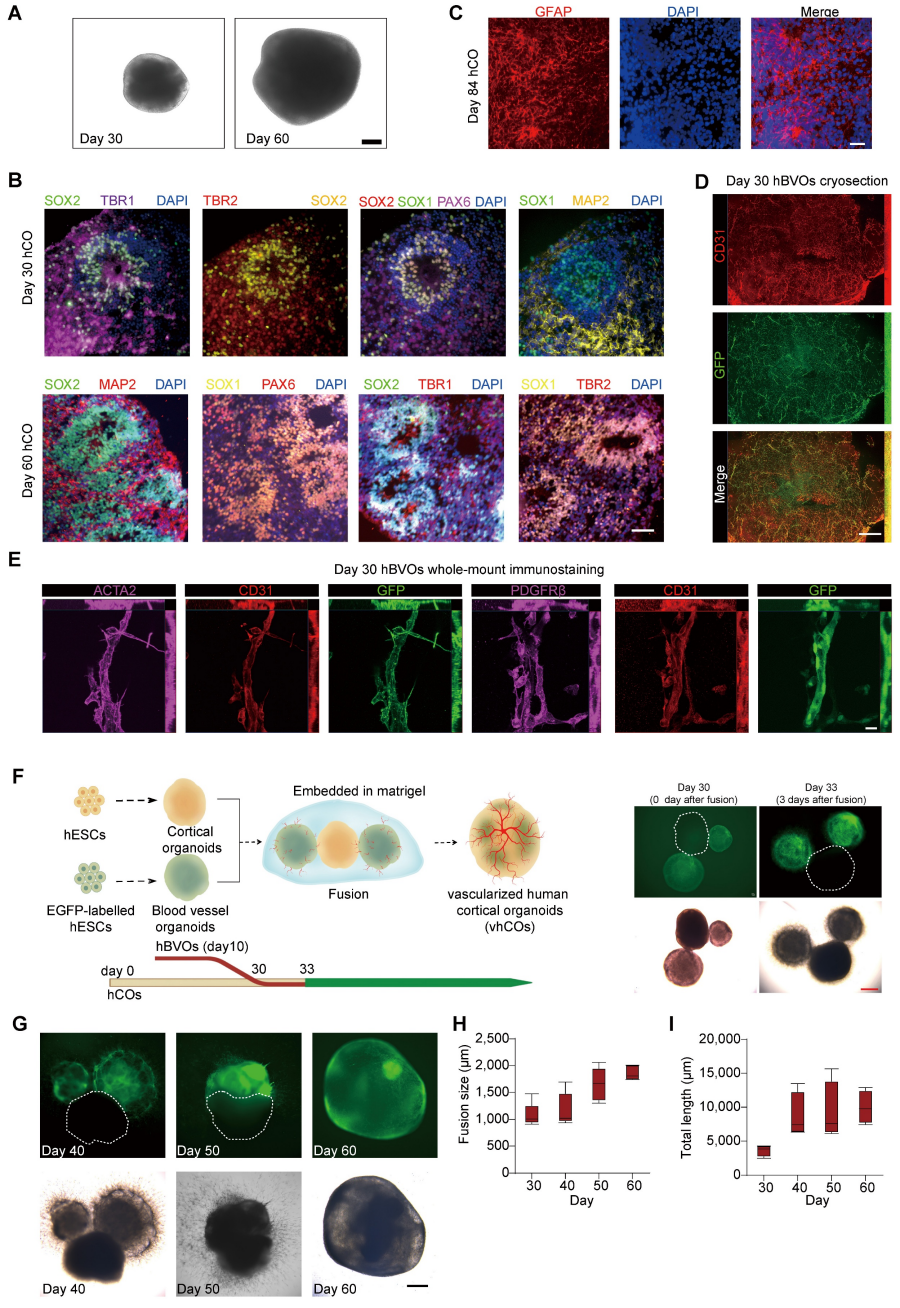
** Generation and characterization of vhCOs. (A)** Bright-field image showing the macroscopic structure of hCOs. Scale bars: 200 μm. **(B)** Confocal microscopy images of hCOs at day 30 and day 60, labeled with cortical layer markers (TBR1), neural progenitor markers (SOX2, SOX1, PAX6), and neuronal maturation markers (MAP2). **(C)** Detection of GFAP-positive astrocytes within day 84 hCOs. Scale bars: 200 μm. **(D)** Cryosection immunostaining showing prominent co-localization of GFP (green, vascular reporter) and CD31 (red, endothelial marker) with vascular network formation within a hBVO at day 30. Scale bar: 500 μm. **(E)** Whole-mount immunostaining demonstrating mural cell-endothelial interactions within vascular structures of hBVOs. Left row: co-alignment of ACTA2 (magenta, smooth muscle cell marker), CD31 (red, endothelial marker), and GFP (green, vascular reporter). Right row: co-alignment of PDGFRβ (magenta, pericyte marker), CD31 (red, endothelial marker), and GFP (green, vascular reporter). Scale bars: 20 μm. Data are representative of n = 4 organoids per group. **(F)** Generation and morphological characterization of vascularized hCOs (vhCOs). Left: Schematic of the fusion protocol combining hCOs and hBVOs to generate vhCOs. Right: Bright-field (bottom) and GFP fluorescence (top) images showing fused hCO-hBVO complexes. Scale bars: 400 μm.** (G)** Morphological validation of hBVO-hCO fusion. Top: GFP fluorescence highlighting hBVO-derived vascular networks (green) integrated with hCOs. Bottom: Corresponding bright-field image of the fused complex (day 40-60). Scale bar: 400 μm. **(H)** Quantitative analysis of fused organoid diameter post-fusion. Data are presented as mean ± SD (n = 4 organoids per group). **(I)** Temporal measurement of vascular network length within fused organoids at sequential developmental stages measured by AngioTool. See also Figure S1.

Initially, we created hCOs from a hESC line (H9) using an established protocol 16, 17 with modifications (Figure S1A), where embryonic bodies (EBs) in suspension grew and differentiated through a series of induction media. Over time, their diameters increased from 419.15 ± 14.40 µm at day 0 to 1556.85 ± 118.98 µm at day 75 (Figure 1A, S1B and S1C). The successful induction of neural cell fate in the hCOs was confirmed by the sequentially regionalized expression of developmental markers: SOX2, SOX1 and PAX6 (neural progenitors), TBR2 (intermediate progenitors [IPs]), TBR1 (cortical layers), and MAP2 (mature neurons) from day 30 to 60. (Figure 1B). Notably, an abundant distribution of GFAP^+^ astrocytes was observed in the hCOs (Figure 1C). Overall, the hCOs induction produced a cortical structure rich in mature astrocytes.

Subsequently, we generated EGFP-labeled hBVOs from *AAVS-EGFP* H9 cells using a modified protocol 18 (Figure S1D). After 10 days of growth in suspension culture, the EBs formed spheroids with a diameter of 894.42 ± 65.93 µm (Figure S1E and S1F). When embedded in Matrigel, these organoids were able to sprout blood vessels (Figure S1E), which were composed of CD31^+^ endothelial cells (ECs) wrapped by PDGFRβ^+^ pericytes and ACTA2^+^ smooth muscle cells (SMCs) (Figure 1D and 1E). Collectively, these results demonstrate the successful induction of hBVOs from hPSCs.

Next, we combined hCOs at day 30 with hBVOs at day 10 together to mimic neuro-vascular co-development (Figure 1F). We placed one hCO and two EGFP-labeled hBVOs together for 3 days, allowing them to adhere firmly to each other (Figure 1F). We then embedded them in a Matrigel droplet and cultured them in a media mixture consisting of half hCO maturation media and half hBVO maturation media to maintain both neural and vascular characteristics (Figure 1G). Over time, EGFP^+^ vessel cells actively invaded the growing hCOs (Figure 1G), indicating ongoing angiogenesis. The hBVOs gradually vascularized the hCOs by extending CD31^+^ endothelial tubes and finally merged into a single organoid with a diameter of 1863.22 ± 134.14 µm within 30 days (Figure 1G-1I). This suggests the formation of capillary networks within the hCOs, which we termed vhCOs.

### vhCOs displayed BBB-like structure

**Figure 2:**
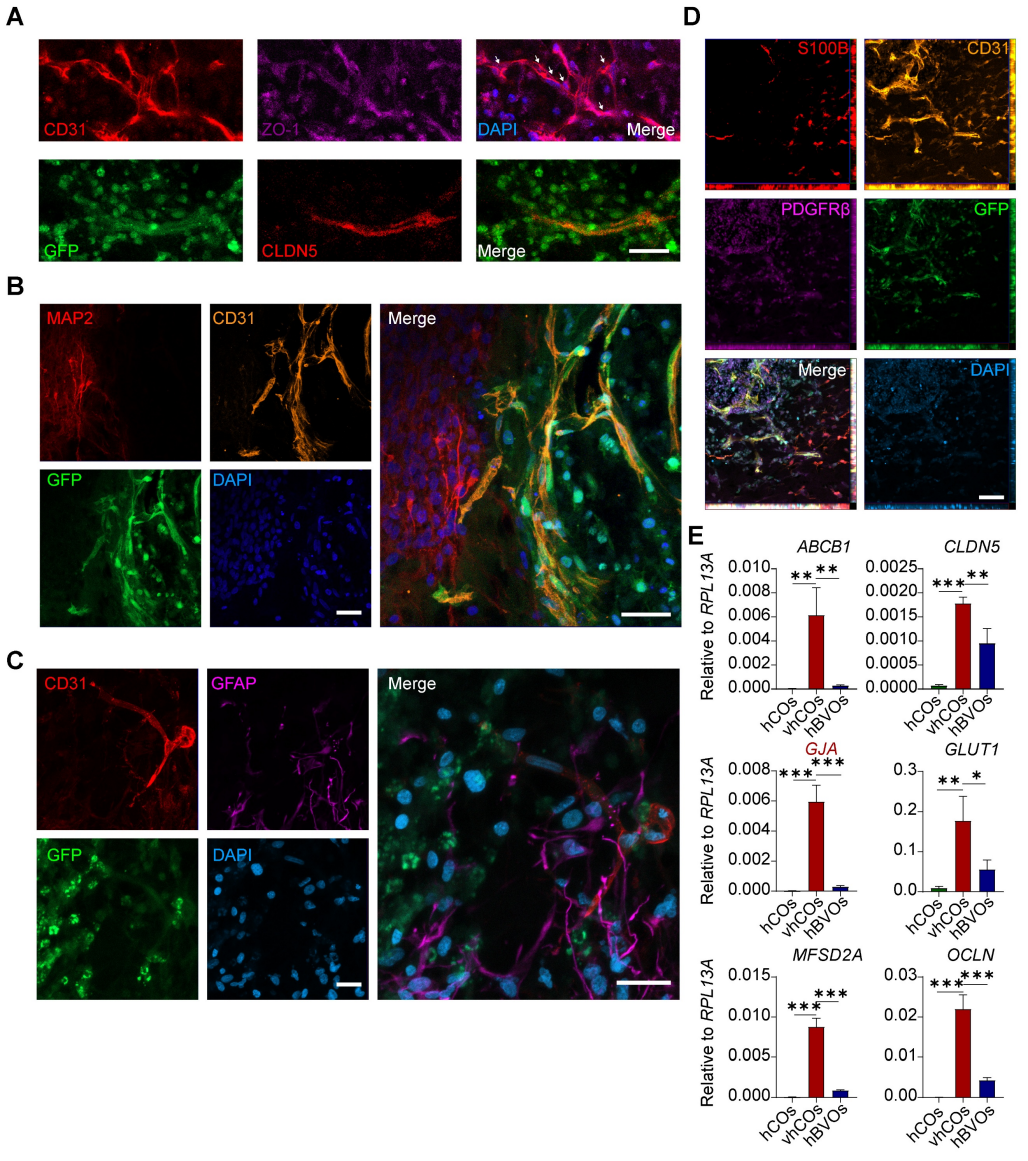
** Characterization of BBB structure in vhCOs. (A)** Immunofluorescence showing expression of BBB markers ZO-1 and CLDN5 by ECs in day 60 vhCOs (30 days post-fusion; n = 3 cultures). Arrows indicate junction-like structure. Scale bar: 20 μm. **(B-D)** Immunofluorescence showing co-alignment of **(B)** MAP2^+^ neurons and CD31^+^ ECs, **(C)** GFAP^+^ astrocytes and CD31^+^ ECs, **(D)** S100B^+^ astrocytes, CD31^+^ ECs, PDGFRβ^+^ pericytes in day 60 vhCOs (30 days post-fusion; n = 3 cultures), respectively. Scale bar: 20 μm. **(E)** RT-qPCR analysis showing expression levels of BBB markers in hCOs at day 50, hBVOs at day 10, and vhCOs at day 60 (30 days post-fusion with hBVOs). Data are presented as mean ± SD (n = 3 technical replicates, each consisting of 3-5 pooled organoids per group).

We next investigated the BBB features of vhCOs. In human embryonic brain development, ECs form capillaries and BBB with tight junctions. These structures were surrounded by astrocytic processes and pericytes for stabilization, and are innervated by neurons and progenitor cells 14. Immunostaining revealed the expression of tight junction markers ZO-1 and CLDN5 in ECs (Figure 2A). The co-alignment of GFAP^+^/S100B^+^ astrocytes, CD31^+^ ECs, PDGFRβ^+^ pericytes, and MAP2^+^ neurons suggested the presence of a typical BBB-like structure (Figure 2B-D). Moreover, RT-qPCR demonstrated abundant expression of BBB markers in the vhCOs:* ABCB*1 (encoding P-glycoprotein), *CLDN5*, *GJA* (astrocytic endfeet marker), *GLUT1* (encoding a facilitative glucose transporter), *MFSD2A* (encoding a membrane transport protein), and *OCLN* than hCOs and hBVOs (Figure 2E). Collectively, these results indicate that vhCOs possess key features of BBB in terms of marker expression and structure.

### Modeling fetal hypoxia-reoxygenation using vhCOs

**Figure 3:**
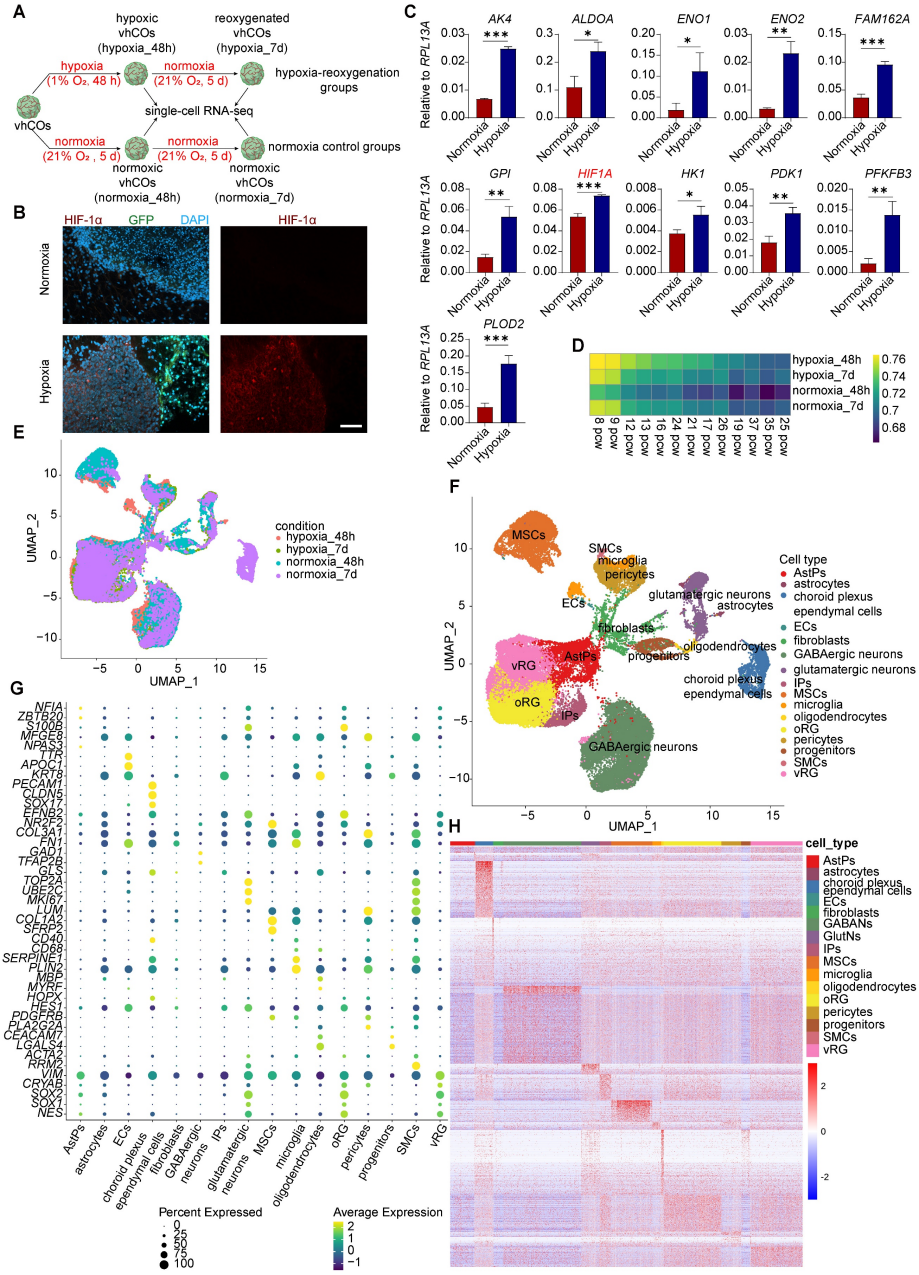
** Single-cell transcriptome analysis reveals diverse cell types in vhCOs. (A)** Schematic illustration of scRNA-seq of normoxic, hypoxic and reoxygenated vhCOs. **(B)** Immunofluorescence showing HIF-1α expression in normoxic (normoxia_48h) and hypoxic (hypoxia_48h) vhCOs (n = 3 cultures). Scale bar: 200 μm. **(C)** RT-qPCR of hypoxic response genes in normoxic (normoxia_48h) and hypoxic (hypoxia_48h) vhCOs. Data are presented as mean ± SD (n = 3 technical replicates, each consisting of 3-5 pooled organoids per group). **(D)** Neurodevelopmental stages estimation of normoxic (normoxia_48h/7d), hypoxic (hypoxia_48h) and reoxygenated (hypoxia_7d) vhCOs by pseudo-bulk analysis of scRNA-seq data. pcw, post-conceptional weeks. See also Figure S2D-2H and S6. **(E and F)** UMAP visualization of the single-cell transcriptome of vhCOs (n= 46,418 cells, 3-pooled organoids for each condition), colored by **(E)** conditions and **(F)** cell types. See also Figure S3A. **(G)** Dot plots displaying cell type-specific gene expression to each cell cluster. vRG, ventricular radial glia; oRG, outer radial glia. See also Figure S3B and S4. **(H)** Heatmap showing DEGs across the 16 cell clusters, each column representing a single cell. See also Table S2 and Figure S5.

To model fetal brain hypoxia-reoxygenation injury, vhCOs at day 60 were subjected to hypoxia (1% O₂, 48 h) followed by 5 days of reoxygenation (21% O₂), while control groups remained under normoxia (21% O₂) (Figure 3A). Immunostaining revealed pronounced upregulation of HIF-1α in hypoxic vhCOs compared to normoxic controls (Figure 3B). Moreover, RT-qPCR confirmed that 48-h hypoxia induced transcriptional activation of key hypoxia-response genes, including *AK4*, *ALDOA*, *ENO1*, *ENO2*, *FAM162A*, *GPI*, *HIF1A*, *HK1*, *PDK1*, *PFKP*, and *PLOD2* (Figure 3C). These findings collectively validate the efficacy of our hypoxia-reoxygenation protocol in recapitulating fetal hypoxic brain injury.

### Dissecting cell types in vhCOs through single-cell transcriptomic analysis

We next performed scRNA-seq on pooled vhCOs under hypoxic (day 62, hypoxic_48h, n=3 organoids), normoxic (day 62/67, normoxic_48h/7d, n=3 organoids each), and reoxygenated (day 67, hypoxic_7d, n=3 organoids) conditions using droplet-based 3' sequencing respectively. After quality filtering (mitochondrial genes >30%, <200 genes/cell, or <3 cells/gene), we retained 46,418 cells with a median of 28,356 genes and UMIs per cell (Figure S2A-C).

We then utilized Spearman's rank correlation coefficient (SRCC) to compare the transcriptome of hypoxic, reoxygenated, and normoxic vhCOs with primary human tissues in Brainspan dataset 19. Our results showed that the transcriptional profiles of these vhCOs were similar to those of the fetal brain tissues ranging from 8 to 16 weeks post gestation (Figure 3D and S2D-H and S6), which corresponds to early mid-gestation period 20.

Next, we projected all single cells of hypoxic, reoxygenated, and normoxic vhCOs onto a UMAP plot and identified 36 distinct clusters (Figure 3E and S3A). By analyzing the expression of marker genes, we annotated these clusters into 16 cell types (Figure 3F). Specifically, clusters 1, 3, 5, 10, 21, 22, 23, 24 and 26 were enriched in radial glia (RG)-specific gene expression, such as *SOX2*, *SOX*1 and *NES* 21 (Figure 3G, S3B and S4); among these clusters, clusters 5, 10, 21, 23, 24 and 26 expressed higher ventricular RG (vRG) marker *VIM* and *CRYAB* 22 compared to clusters 1, 3, and 22, which enriched in outer RG (oRG) marker *HOPX* and *TNC* 23 (Figure 3G, S3B and S4).

Therefore, we annotated clusters 5, 10, 21, 23, 24 and 26 as vRG and clusters 1, 3 and 22 as oRG. Cluster 9 was annotated as IPs due to its enriched expression of proliferating markers including *TOP2A*, *UBE2C* and *MKI67* 24 (Figure 3G, S3B and S4). Clusters 6 and 20 were annotated as AstPs, as it showed higher upregulation of gliogenesis-related genes *NFIA* and *ZBTB20* 25 and weak expression of stem cell gene *SOX2* and *SOX1* (Figure 3G, S3B and S4). Clusters 2, 4, 7 and 8 represented GABAergic neurons that expressed *GAD1* and *TFAP2B* 26 (Figure 3G, S3B and S4). Clusters 11 and 19 were annotated as glutamatergic neurons, expressing *GLS* 27 (Figure 3G, S3B and S4). In terms of non-neural cells, oligodendrocytes were identified as cluster 29 expressing *MBP* and *MYRF* (Figure 3G, S3B and S4); microglia were identified as clusters 25 and 27, highly expressing *SERPINE1* and *PLIN2* 28 (Figure 3G, S3B and S4); cluster 14 was unidentified progenitors expressing *CEACAM7* and *LGALS4* (Figure 3G, S3B and S4); mesenchymal stromal cells (MSCs) resided in clusters 0, 17, 28 and 30, expressing *COL1A2*, *SFRP2* and *LUM* 29 (Figure 3G, S3B and S4); key components of BBB included astrocytes (clusters 33 and 35) expressing *S100B* and *MFGE8* 30, ECs (cluster 34) expressing *PECAM1* and *CDH5* 31, and pericytes (clusters 12 and 13) expressing* PDGFRB* and *PLA2G2A* 32 (Figure 3G, S3B and S4). Interestingly, ECs expressed arterial marker *SOX17* and *EFNB2* but not venous marker *NR2F2* (Figure 3G, S3B and S4), indicating arterial characteristics of these ECs within vhCOs. Other blood vessel components, SMCs, were also identified in cluster 23, expressing *ACTA2* and *RRM2* 31 (Figure 3G, S3B and S4). Notably, clusters 15 and 16 were identified as choroid plexus ependymal cells, expressing *TTR*, *APOC1* and* KRT8* 33, which were only found in normoxic vhCOs at day 67 (Figure 3E-G, S3A-B and S4).

To further characterize these cell populations, we performed differential expression gene (DEG) analysis. The DEG heatmap revealed cell type-specific expression patterns (Figure 3H). Gene Ontology (GO) enrichment analysis of these DEGs showed conserved biological functions corresponding to annotated cell population, with gene set expression profiles matching their *in vivo* counterparts (Table S2 and Figure S5). vRGs were characterized by enriched expression of GO terms related to neuronal stem cell maintenance and proliferation. oRGs exhibited enriched expression of GO terms associated with neuronal and glial differentiation. IPs predominantly expressed GO terms linked to cell division, while AstPs focused on astrocyte differentiation. GABAergic and glutamatergic neurons highly expressed genes related to neural projection development. Oligodendrocytes enriched lipid metabolic and membrane genes. ECs displayed functional genes involving vasculogenesis, angiogenesis, and vasculature development. Astrocytes showed S100 protein binding and vesicle organization. Pericytes enriched in collagen and integrin signaling. SMCs expressed actomyosin and contractile actin filament genes. Microglia predominantly expressed lysosome-related genes. MSCs and fibroblasts exhibited typical mesenchymal characteristics. The SRCC further demonstrated transcriptomic resemblance of these cell types in normoxic, hypoxic and reoxygenated vhCOs to primary fetal brain tissues ranging from 8-21 weeks (Figure S6).

Collectively, we identified the presence of neural cells, such as vRG, oRG, IPs, AstPs, GABAergic neurons, glutamatergic neurons, and non-neural cells such as astrocytes, ECs, pericytes, SMCs, microglia, MSCs, fibroblasts, within vhCOs, which exhibit transcriptomic similarities to primary human fetal brain tissues at early to midgestation stages.

### Lineage-specific hypoxia signaling and metabolic reprogramming in vhCOs exposed to hypoxia-reoxygenation

**Figure 4:**
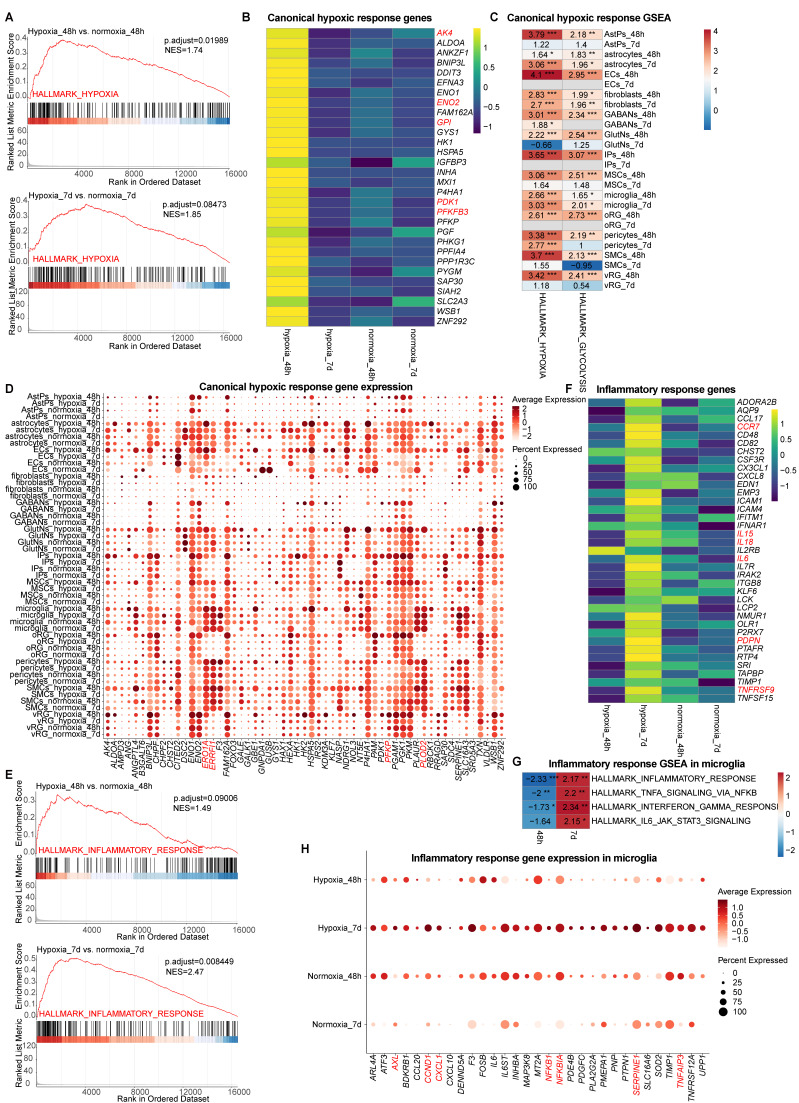
** Characterization of canonical hypoxic and inflammatory response within vhCOs during hypoxia-reoxygenation. (A)** Pseudo-bulk GSEA of scRNA-seq data in hypoxic (hypoxia_48h) and reoxygenated (hypoxia_7d) vhCOs vs. normoxic (normoxia_48h/7d) vhCOs, respectively, in terms of HALLMARK_HYPOXIA. **(B)** Heatmap showing the expression of hypoxic response genes by pseudo-bulk analysis of scRNA-seq data in hypoxic (hypoxia_48h), reoxygenated (hypoxia_7d) vhCOs and normoxic (normoxia_48h/7d) vhCOs, respectively. **(C)** Heatmap showing GSEA of NES of indicated gene sets (HALLMARK_HYPOXIA, HALLMARK_GLYCOLYSIS) across 13 cell types in DEGs of in hypoxic (hypoxia_48h) and reoxygenated (hypoxia_7d) vhCOs vs. normoxic (normoxia_48h/7d) vhCOs, respectively. NES, normalized enrichment score; *padj<0.05; **padj<0.01; *** *padj*<0.001. **(D)** Dot plots showing hypoxic response and glycolysis-related gene expression in all cell population of normoxic (normoxia_48h/7d), hypoxic (hypoxia_48h) and reoxygenated (hypoxia_7d) vhCOs. **(E)** Pseudo-bulk GSEA of scRNA-seq data in hypoxic (hypoxia_48h) and reoxygenated (hypoxia_7d) vhCOs vs. normoxic (normoxia_48h/7d) vhCOs, respectively, in terms of HALLMARK_INFLAMMATORY_RESPONSE. **(F)** Heatmap showing the expression of inflammatory response genes by pseudo-bulk analysis of scRNA-seq data in hypoxic (hypoxia_48h), reoxygenated (hypoxia_7d) and normoxic (normoxia_48h/7d) vhCOs, respectively. **(G)** Heatmap showing GSEA of NES of indicated gene sets in DEGs within microglia of hypoxic (hypoxia_48h) and reoxygenated (hypoxia_7d) compared to normoxic (normoxia_48h/7d) vhCOs, respectively. NES, normalized enrichment score; *padj<0.05; **padj<0.01; *** *padj*<0.001. **(H)** Dot plots showing inflammatory response genes expression within microglia of normoxic (normoxia_48h/7d), hypoxic (hypoxia_48h) and reoxygenated (hypoxia_7d) vhCOs. See also Table S3, S4 and S5.

We next investigated canonical hypoxic response during hypoxia-reoxygenation in vhCOs. Pseudo-bulk GSEA of scRNA-seq data demonstrated acute hypoxia-induced upregulation of canonical hypoxia pathways (NES=1.74, *padj*=0.01989) at 48-h post-hypoxia, with subsequent attenuation to marginal increase following 5-day reoxygenation (NES=1.85, *padj*>0.05) (Table S3 and Figure 4A), consistent with previous reports 11, 12. Canonical hypoxia-responsive genes including *AK4*, *ENO2*, *GPI*, *PDK1*, and *PFKFB3 etc.* exhibited transient induction during hypoxia that normalized post-reoxygenation (Figure 4B), in line with RT-qPCR results (Figure 3C). Notably, cell-type specific responses emerged: most neural cells (except from GABAergic neurons) showed acute transcriptional activation of canonical hypoxia-responsive genes confined to hypoxia exposure (hypoxia_48h), while most non-neural populations (astrocytes, microglia, pericytes, and fibroblasts) maintained elevated hypoxia signatures through hypoxia-reoxygenation (Table S4-5 and Figure 4C). All cell types shifted toward glycolytic metabolism during 48-h acute hypoxia, showing increased expression of glycolysis regulators (*ERO1A*,* ERRFI1*, *PFKP1*,* PLOD2*, *GPI etc.*); however, sustained upregulation after 5 days post-reoxygenation was exclusive to non-neural populations like astrocytes, microglia, pericytes, and fibroblasts (Figure 4C and 4D).

Together, these findings reveal a heterogeneity in canonical hypoxic response across different cell types in vhCOs undergoing hypoxia-reoxygenation.

### Hypoxia-reoxygenation drove biphasic inflammatory activation in vhCOs

Consistent with established hypoxia-reoxygenation pathology in premature brain 7, pseudo-bulk GSEA of scRNA-seq data revealed a global progressive activation of inflammatory pathways in vhCOs, transitioning from marginal induction at 48-h post-hypoxia (NES=1.49, *padj*>0.05) to robust upregulation following 5-day reoxygenation (NES=2.47, *padj*<0.01) (Table S3 and Figure 4E). Key inflammatory mediators (*CCR7*, *IL15*, *IL8*, *IL6*, *PDPN*, and *TNFRSF9*) demonstrated delayed activation, achieving significance exclusively post-reoxygenation (Figure 4F). As microglia were the sole immune cells in vhCOs, we examined inflammation-related gene sets expression in microglia. Interestingly, GSEA of microglia uncovered a temporal phenotypic shift: acute hypoxia (hypoxia_48h) suppressed canonical inflammatory pathways (inflammatory response, TNF signaling via NF-kB, interferon gamma response), while reoxygenation (hypoxia_7d) triggered their hyperactivation (Table S4-5 and Figure 4G). Coordinated upregulation of activation markers (*AXL*, and *SERPINE1*) and inflammatory regulators (*CCND1*, *CXCL1*, *NFKB1*, *NFKBIA*, and *TNFAIP3*) specifically marked the pro-inflammatory transition after 5-day reoxygenation (Figure 4H). Collectively, these findings define a microglial state-switching mechanism underlying delayed inflammation during hypoxia-reoxygenation.

### AstPs were most sensitive to hypoxia-reoxygenation among neural-lineage cell types in vhCOs

**Figure 5:**
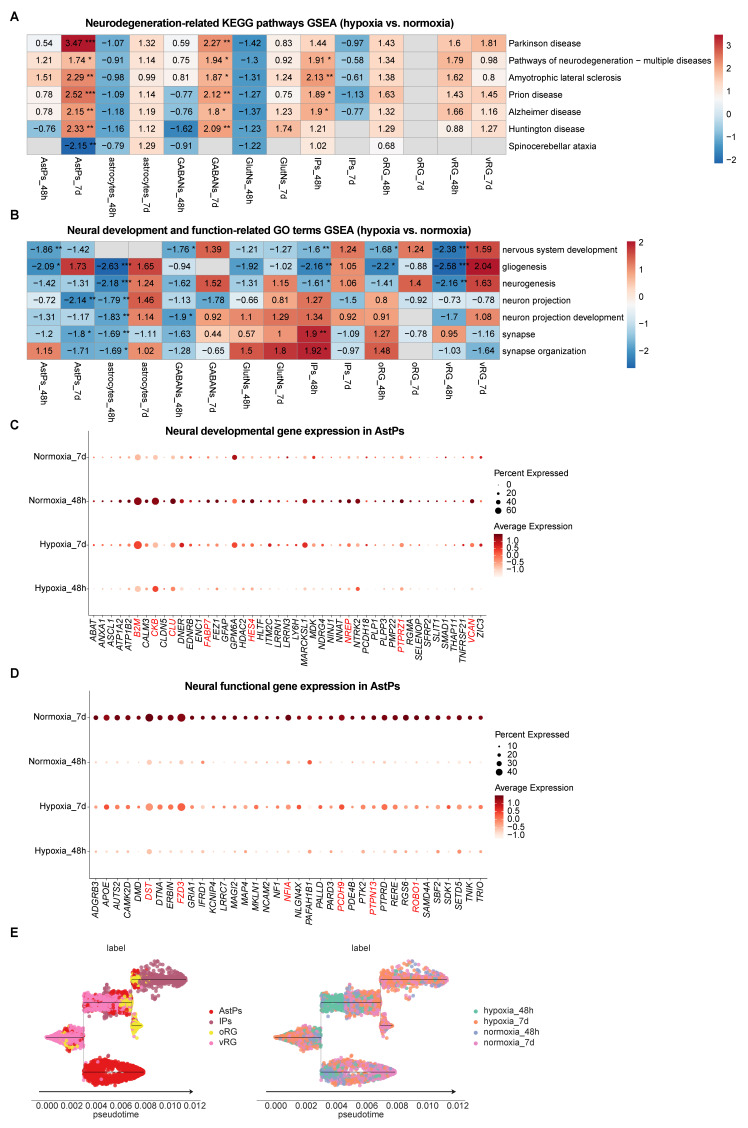
** Distinct responses among neural-lineage cell types within vhCOs during hypoxia-reoxygenation. (A)** Heatmap showing GSEA of NES of indicated neurodegenerative disease-associated KEGG pathways across indicated neural-lineage cell types in DEGs of hypoxic (hypoxia_48h) and reoxygenated (hypoxia_7d) vhCOs compared to normoxic (normoxia_48h/7d) vhCOs, respectively. NES, normalized enrichment score; *padj<0.05; **padj<0.01; *** *padj*<0.001. **(B)** Heatmap showing GSEA of NES of indicated neural developmental and functional GO terms across indicated neural-lineage cell types in DEGs of hypoxic (hypoxia_48h) and reoxygenated (hypoxia_7d) vhCOs compared to normoxic (normoxia_48h/7d) vhCOs, respectively. NES, normalized enrichment score; *padj<0.05; **padj<0.01; *** *padj*<0.001. **(C and D)** Dot plots showing (C) neural developmental and **(D)** functional genes expression within AstPs of normoxic (normoxia_48h/7d), hypoxic (hypoxia_48h) and reoxygenated (hypoxia_7d) vhCOs. **(E)** Stream visualization of developmental trajectories of indicated neural progenitor cell types in vhCOs, colored by cell types and conditions. See also Table S4, S5, and Figure S7A and C.

To resolve cell-type-specific responses during hypoxia-reoxygenation, we conducted DEG analysis across hypoxic, normoxic and reoxygenated vhCOs (Table S4 and Figure S7A-B). GSEA of of KEGG pathways revealed sustained activation of neurodegeneration-associated pathways—Parkinson disease, Pathways of neurodegeneration-multiple diseases, Amyotrophic lateral sclerosis, Prion disease, Alzheimer disease, and Huntington disease—in AstPs and GABAergic neurons throughout hypoxia-reoxygenation (Table S5 and Figure 5A). In contrast, other neural-lineage cells exhibited only transient activation of these pathways at 48 h post-hypoxia or showed minimal changes (Table S5 and Figure 5A). Specifically, these populations specifically maintained elevated oxidative stress markers (*ATP5PF*, *COX4I1*, *COX6C*, *COX7A2L*, *COX7C*, *NDUFA2*, and *NDUFB11 etc.*) across hypoxia-reoxygenation (Figure S7C).

To detailly figure out the developmental and functional deficits caused by hypoxia-reoxygenation across different neural-lineage cell types, we compared gene sets expression that were related to neural development and function. GSEA of GO term showed transient suppression but recovery of neurogenic programs (including nervous system development, gliogenesis, and neurogenesis) in all progenitors except AstPs, which failed to recover post-reoxygenation (Table S5 and Figure 5B). While function-associated GO terms (neuron projection, neuron projection development, synapse and synapse organization) in mature astrocytes and neurons exhibited transient downregulation with recovery, AstPs displayed persistent suppression of these genes (Table S5 and Figure 5B), corroborated by corresponding key gene expression patterns (*B2M*, *CKB*, *CLU*, *FABP7*, *HES4*, *NREP*, *PTPRZ1*, *VCAN*, *DST*, *FZD3*, *NFIA*, *PCDH9*, *PTPN13*, *ROBO1 etc.*) (Figure 5C and 5D). Pseudotime trajectory analysis further confirmed developmental delay specific to AstPs during reoxygenation (Figure 5E).

Together, these results suggest that AstPs were most sensitive to hypoxia-reoxygenation, characterized by chronic neurodegenerative pathway activation, developmental arrest and impaired synaptic integrity.

### Non-neural cells drove compartmentalized vascular remodeling in vhCOs during hypoxia-reoxygenation

**Figure 6:**
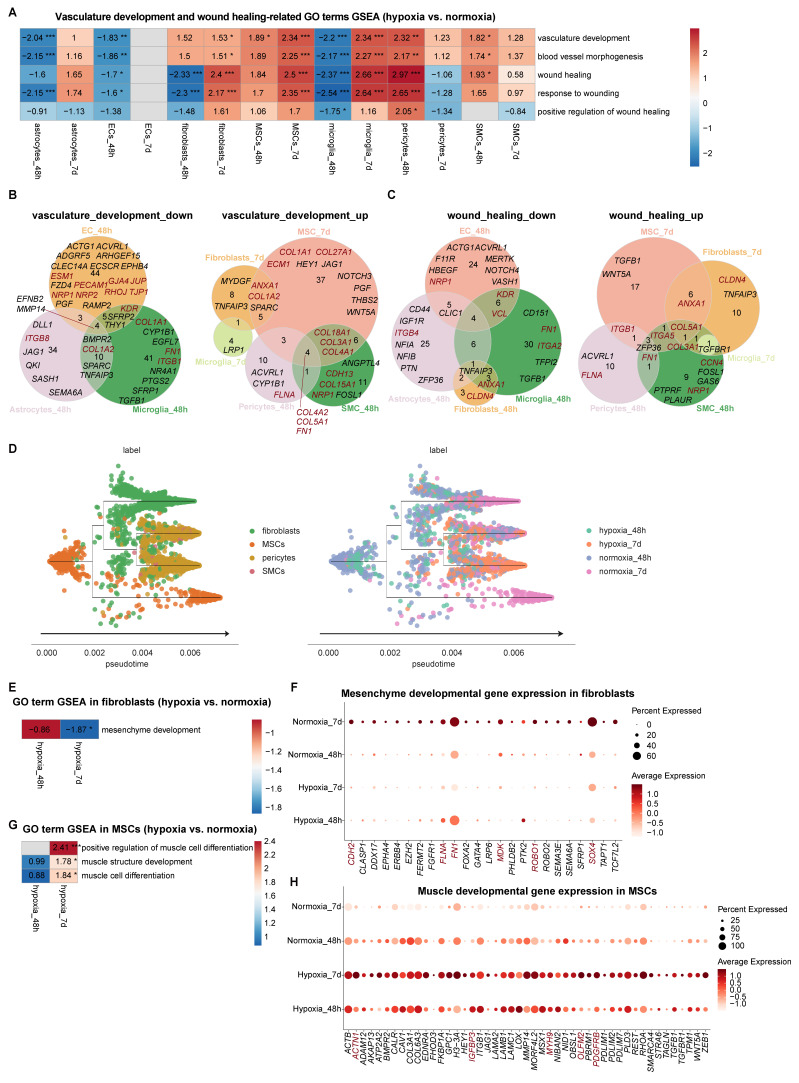
** Distinct responses among non-neural cells within vhCOs during hypoxia-reoxygenation. (A)** Heatmap showing GSEA of NES of indicated vasculature remodeling-associated gene sets across indicated non-neural cells in DEGs of hypoxic (hypoxia_48h) and reoxygenated (hypoxia_7d) vhCOs compared to normoxic (normoxia_48h/7d) vhCOs, respectively. NES, normalized enrichment score; *padj<0.05; **padj<0.01; *** *padj*<0.001. **(B and C)** Venn diagrams showing upregulated and downregulated DEGs of **(B)** vasculature development and **(C)** wound healing within indicated non-neural cells of hypoxic (hypoxia_48h) and reoxygenated (hypoxia_7d) vhCOs compared with that of normoxic (normoxia_48h/7d) vhCOs, respectively. **(D)** Stream plots visualizing developmental trajectories of MSC-lineage cells, colored by cell types and conditions, respectively. **(E)** Heatmap showing GSEA NES of mesenchyme development gene set in DEGs within fibroblasts of hypoxic (hypoxia_48h) and reoxygenated (hypoxia_7d) vhCOs compared to normoxic (normoxia_48h/7d) vhCOs, respectively. NES, normalized enrichment score; *padj<0.05; **padj<0.01; *** *padj*<0.001. **(F)** Dot plots showing mesenchyme developmental genes expression within fibroblasts of normoxic (normoxia_48h/7d), hypoxic (hypoxia_48h) and reoxygenated (hypoxia_7d) vhCOs. **(G)** Heatmap showing GSEA of NES of indicated gene sets in DEGs within MSCs of hypoxic (hypoxia_48h) and reoxygenated (hypoxia_7d) vhCOs compared to normoxic vhCOs, respectively. NES, normalized enrichment score; *padj<0.05; **padj<0.01; *** *padj*<0.001. **(H)** Dot plots showing muscle developmental genes expression within MSCs of normoxic (normoxia_48h/7d), hypoxic (hypoxia_48h) and reoxygenated (hypoxia_7d) vhCOs. See also Table S3, S4, S5, and Figure S7B.

GSEA of vascular remodeling pathway regarding vasculature development (vasculature development, blood vessel morphogenesis) and repairment (wound healing, response to wounding) uncovered compartmentalized responses among non-neural cells during hypoxia-reoxygenation in vhCOs (Table S5 and Figure 6A). At early-phase response (48-h post-hypoxia), ECs, microglia, and astrocytes suppressed vascular development (downregulation of pro-angiogenesis genes *EFNB2* and *MMP14*), with barrier integrity genes (*GJA4*, *JUP*, *RHOJ* and *TJP1*) and signaling receptors (*NRP1*, *NRP2*, *PECAM1*, *ESM1* and *KDR*) downregulated in ECs, indicating BBB destabilization and EC function impairment (Figure 6A-B); meanwhile, pericytes and SMCs upregulated vasculature and wounding repair pathways (Figure 6A-C). At late-phase adaptation (5-day reoxygenation), MSCs and fibroblasts exhibited pro-angiogenic activation with upregulation of Notch signaling molecules (*HEY1*, *JAG1*, *NOTCH3 etc.*); microglia transitioned from barrier-disruptive to remodeling-promoting phenotypes (Figure 6A-C). Dynamic extracellular matrix (ECM) reorganization accompanied these changes, with expression fluctuations of genes encoding collagen (*COL1A1*, *COL1A2*, *COL3A1*, *COL4A1*, *COL4A2*, *COL5A1*, *COL15A1*, *COL18A1*, *COL27A1*,* etc.)* and other ECM proteins (*CCN4*, *FN1*, *FLNA*, *ITGA2*, *ITGA5*, *ITGB1*, *ITGB8*, *etc.*) (Figure 6B and 6C).

Given the critical role of MSCs in cerebrovascular development 29, we mapped their lineage commitment dynamics through pseudotime trajectory analysis. Under normoxic conditions, MSCs differentiated into fibroblasts, pericytes and SMCs (Figure 6D). At early-phase response (48-h post-hypoxia), there was minimal shifts in MSC-fibroblast-SMC distribution. At late-phase adaptation (5-day reoxygenation), there was significant reduction in undifferentiated MSCs and mature fibroblasts (Figure 6D). Downregulation of mesenchymal developmental genes (*CDH2*,* FLNA*,* FN1*, *MDK*, *ROBO1*, *SOX4*,* etc.*) likely drove fibroblast attrition (Figure 6E and 6F). Notably, hypoxia-reoxygenation triggered myogenic differentiation in MSCs, evidenced by upregulated muscle-specific regulators (*ACTN1*, *IGFBP3*, *MYH9*, *OLFM2*, *PDGFRB etc.*), which may explain the depletion of undifferentiated MSC pools (Figure 6G and 6H).

Collectively, these results demonstrate compartmentalized responses among non-neural cells during hypoxia-reoxygenation in vhCOs with dynamics of vascular development and ECM gene expression.

### Cellular interaction alteration in vhCOs during hypoxia-reoxygenation

To investigate intercellular communication dynamics during hypoxia-reoxygenation, we applied *CellPhoneDB* 34, a computational tool that infers ligand-receptor interaction networks from single-cell transcriptomic data across all cell clusters (Figure S8A). Among neural lineage interactions, the *PPIA-BSG* pathway—linked to ERK activation 35 — was upregulated in hypoxic and reoxygenated vhCOs (Figure S8B). Conversely, the *PTN-PTPRZ1* interaction, which promotes astrogliosis 36 and neurogenesis 37, 38, decreased under hypoxia but partially recovered after reoxygenation (Figure S8B), possibly explaining transient astrocyte dysfunction (Figure 5B).

**Figure 7:**
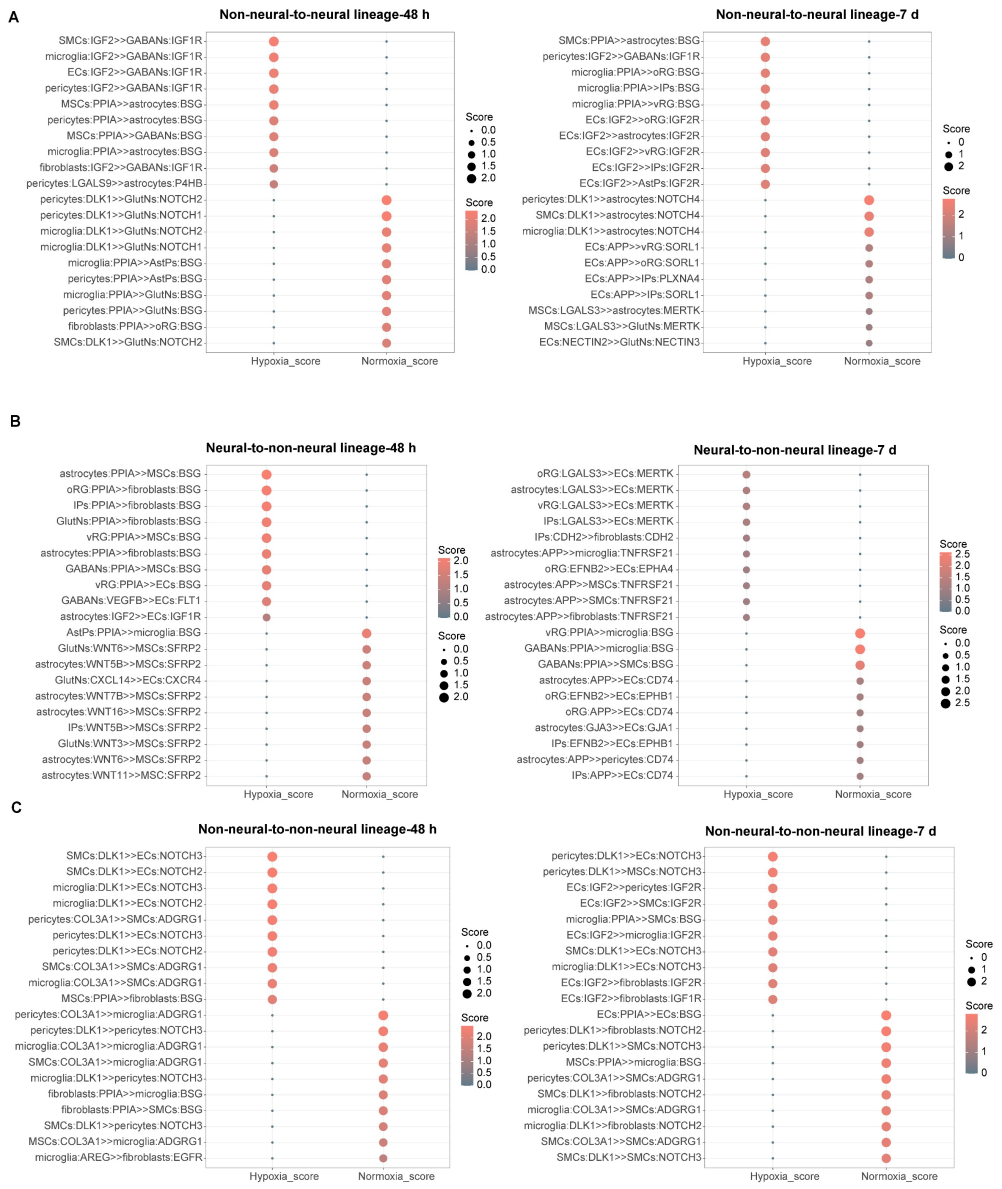
** Characterization of cellular interaction alterations within vhCOs during hypoxia-reoxygenation. (A-C)** *CellPhoneDB*-predicted top 10 ligand-receptor interactions across **(A)** non-neural-to-neural, **(B)** neural-to-non-neural, and **(C)** non-neural-to-non-neural lineages in vhCOs under normoxic (normoxia_48h/7d), hypoxic (hypoxia_48h), and reoxygenated (hypoxia_7d) conditions. Interaction strength is encoded by node size (scaled to scores) and color intensity. See also Figure S8.

For non-neural-to-neural lineage communication, *PPIA-BSG* interactions were upregulated in astrocytes and GABAergic neurons but downregulated in AstPs and glutamatergic neurons at 48-h post-hypoxia, with overall enrichment in reoxygenated vhCOs (Figure 7A). Notably,* IGF2*, a neurogenic factor 39-41, shifted from upregulated *IGF2-IGF1R* to *IGF2-IGF2R* interactions during hypoxia-reoxygenation (Figure 7A), implying non-neural lineage involvement in neurogenesis recovery. Additionally, Notch signaling declined: glutamatergic neurons showed reduced *DLK1-NOTCH1* interactions at 48-h post-hypoxia, and astrocytes exhibited diminished *DLK1-NOTCH4* interactions 5 days post-reoxygenation (Figure 7A).

Within BBB components, ECs exhibited upregulated Notch signaling (*DLK1-NOTCH3/2*) from SMCs, microglia, and pericytes during hypoxia-reoxygenation (Figure 7B and 7C). Although ECs received elevated *LGALS3-MERTK* interactions—which support BBB maintenance 42—from neural progenitors and astrocytes by 5 days post-reoxygenation, tight junctions (*GJA3-GJA1*) with astrocytes showed reduction (Figure 7B and 7C). Concurrently, astrocytes transmitted *APP-TNFRSF21* signals, and ECs secreted *IGF2* to MSCs, SMCs, and fibroblasts at this timepoint (Figure 7B and 7C). Pericytes exhibited diminished *DLK1-NOTCH3* signaling at 48-h post-hypoxia, while SMCs displayed downregulated *ADGRG1* by 5 days post-reoxygenation (Figure 7B and 7C). Other non-neural lineages also showed altered communication: microglia had reduced *PPIA-BSG* interactions, and MSCs/fibroblasts received decreased *Wnts-SFRP2* and *PPIA-BSG* signaling, respectively, from neural cells at 48-h post-hypoxia (Figure 7B-C). Together, these results reveal intricate BBB breakdown-repair mechanisms and non-neural lineage adaptations during hypoxia-reoxygenation.

### GABAergic neuron subtypes exhibited divergent responses to hypoxia-reoxygenation in vhCOs

While animal models have documented GABAergic neuron impairment following hypoxia-reoxygenation 43, 44, our vhCO model maintained overall neuronal integrity (Figure 5B), prompting investigation of subtype-specific responses.

**Figure 8:**
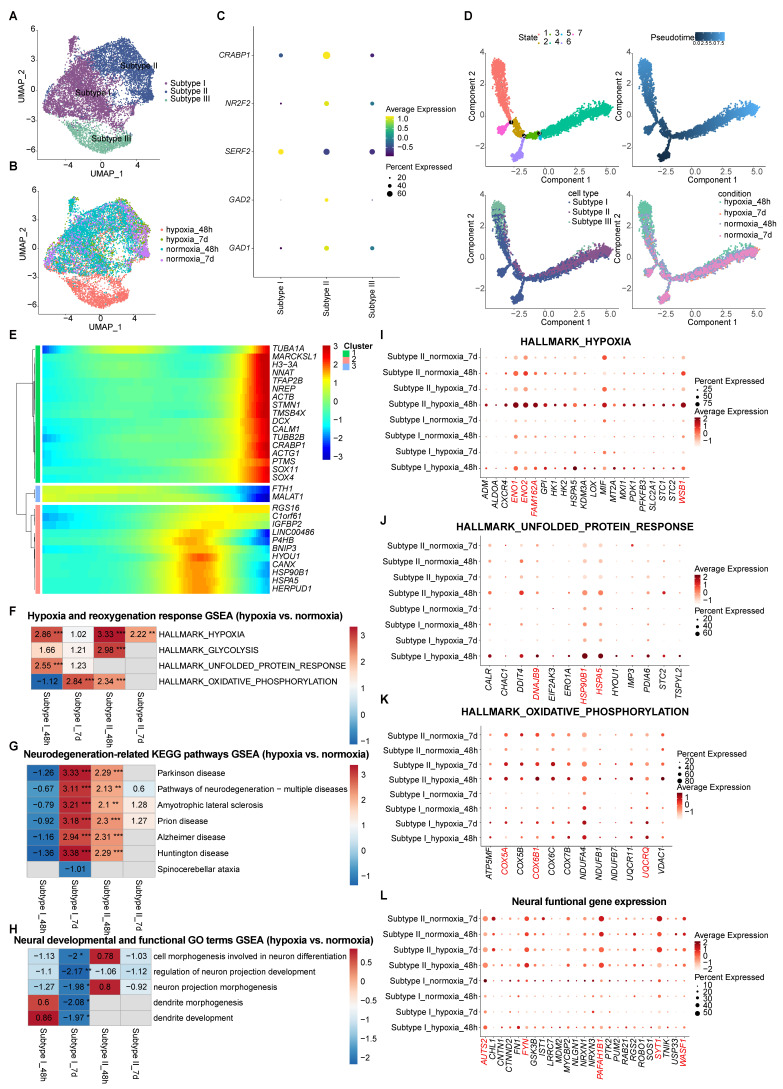
** Characterization of transcriptomic dynamics of GABAergic neuron subtypes during hypoxia-reoxygenation. (A and B)** UMAP visualization of the single-cell transcriptome of GABAergic neurons (n=10,247 cells) within vhCOs, colored by **(A)** cell clusters and **(B)** conditions. See also Figure S9A-C. **(C)** Dot plots displaying subtype-specific gene expression to each cell cluster within GABAergic neurons. See also Figure S9A-E. **(D)** Monocle pseudotime analysis delineating developmental trajectories of GABAergic neurons in vhCOs. **(E)** Heatmap showing the expression level of top 30 DEGs along pseudotime within GABAergic neurons of vhCOs. **(F-H)** Heatmap showing GSEA of NES of indicated gene sets in DEGs within GABAergic neuron Subtype I and II of hypoxic (hypoxia_48h), and reoxygenated (hypoxia_7d) vhCOs compared to normoxic (normoxia_48h/7d) vhCOs, respectively. NES, normalized enrichment score; *padj<0.05; **padj<0.01; *** *padj*<0.001. **(I-L)** Dot plots showing indicated gene expression within GABAergic neuron Subtype I and II of normoxic (normoxia_48h/7d), hypoxic (hypoxia_48h) and reoxygenated (hypoxia_7d) vhCOs. See also Table S6, S7 and Figure S9.

UMAP clustering delineated three transcriptionally distinct GABAergic subtypes (Figure 8A and 8B), each defined by unique marker expression (Figure 8C, S9A and S9B) and GO term enrichment profiles (Table S6 and Figure S9C-E): Subtype I exhibited low *GAD1*/*GAD2* expression and a translationally active state (Figure S9C), suggesting a progenitor-like phenotype; Subtype II displayed elevated *GAD1* and *GAD2* levels, alongside enriched neural function genes including axon genesis and synapse formation (Figure S9D), indicative of mature neuron activity; Subtype III showed intermediate *GAD1* expression with an endoplasmic reticulum (ER)-stress signature (Figure S9E), reflecting acute cellular stress responses. Notably, Subtype III was predominantly detected under hypoxia, while Subtypes I and II persisted across all experimental groups (Figure 8B).

Pseudotime trajectory analysis identified a maturation continuum, with Subtype I representing immature precursors and Subtype II mature neurons. Migration and neurite development genes (*TUBA1A, MARCKSL1, NNAT, TFAP2B, NREP, ACTB, STMN1*, *etc.*) peaked in late-pseudotime state 3 (Figure 8D, 8E and S9F-H). Subtype III, exclusive to 48-h hypoxia, diverged from this trajectory, indicating hypoxia-induced disruption (Figure 8D). Consistent with this, state 1—enriched for Subtype III—showed peaked expression of ER-stress genes (*BNIP3, HYOU1, CANX, HSPA5, HERPUD1*, *etc*) (Figure 8D, 8E and S9F-H). Subtype distributions reverted to normoxic patterns after 5-day reoxygenation (hypoxia_7d), suggesting Subtype III reflects a transient adaptive state rather than permanent reprogramming (Figure 8D). In contrast, Subtypes I and II persisted as stable GABAergic neuron populations, marking them as robust candidates for further mechanistic study.

GSEA further revealed divergent hypoxia-reoxygenation dynamics between Subtypes I and II (Table S7 and Figure 8F-L). In Subtype I, acute 48-h hypoxia triggered hypoxic (*ENO1*, *ENO2*, *FAM162A*, *WSB1*, *etc.*) and unfolded protein response (UPR) including *DNAJB9*, *HSP90B1*, *HSPA5*, *etc.*; 5-day reoxygenation upregulated oxidative phosphorylation (*COX5A*, *COX6B1*, *UQCRQ*, *etc.*) and neurodegenerative-associated genes, and suppressed neural development and function gene expression (*AUTS2*, *FYN*, *PAFAH1B1*, *SYT1*, *WASF1*, *etc.*) (Figure 8F-L). In Subtype II, there was sustained hypoxia signaling with transient oxidative phosphorylation activation (*padj* < 0.01 at 48-h hypoxia), accompanied by little change of UPR gene expression; despite upregulated at 48-h post-hypoxia, neurodegenerative-associated genes normalized post-reoxygenation, accompanied by little change of neural development and function gene expression (Figure 8F-L). Together, these findings identify Subtype I as the vulnerability hotspot, exhibiting prolonged functional deficits, while Subtype II demonstrates resilience to hypoxia-reoxygenation stress.

**Figure 9:**
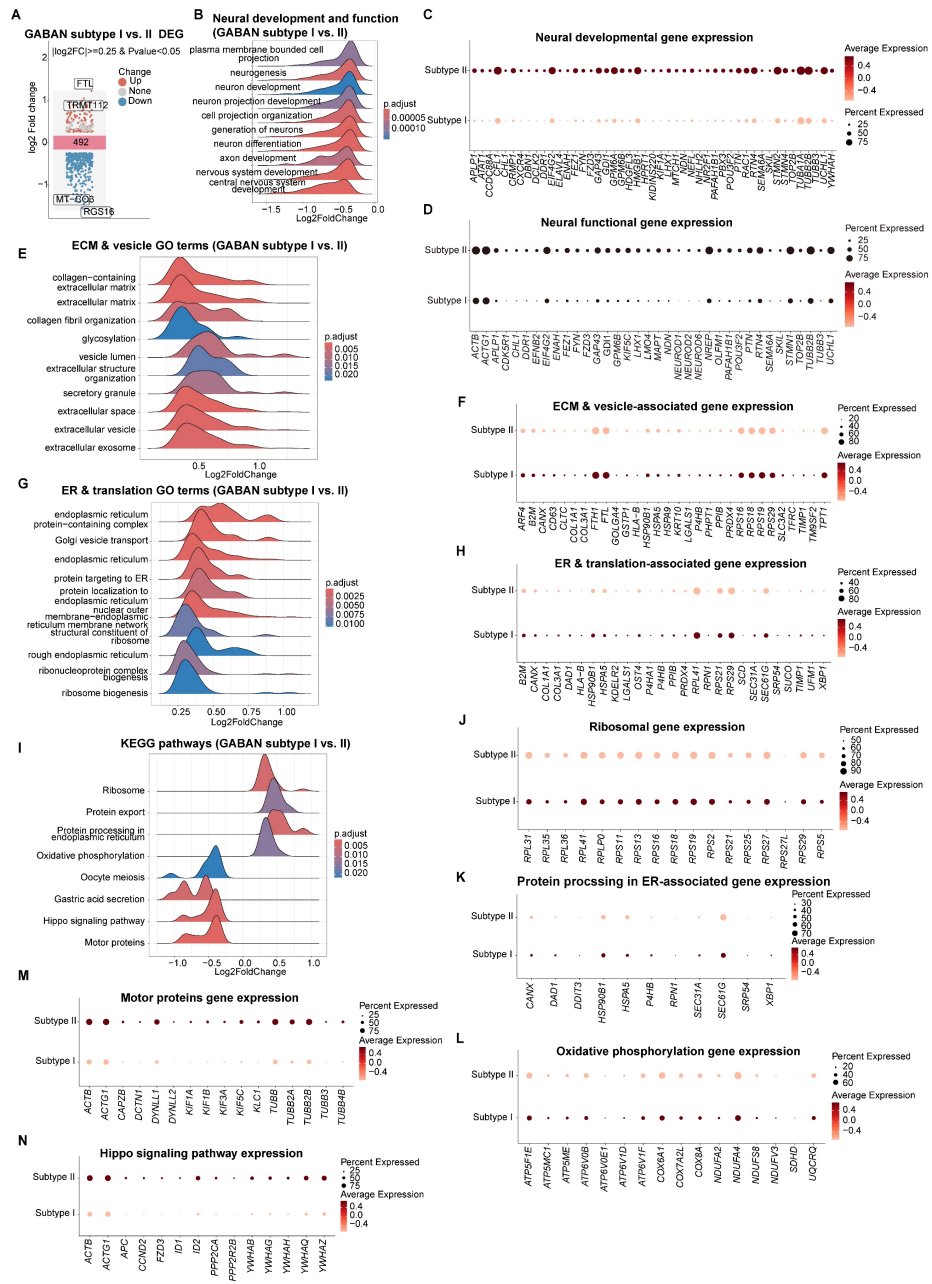
** Characterization of transcriptomic differences between GABAergic neuron subtypes I and II. (A)** Volcano plots displaying DEGs from GABAergic neuron type I to type II in vhCOs. Top ranked genes are shown. **(B, E, G and I)** GSEA plots showing NES of indicated gene sets in DEGs within GABAergic neuron Subtype I vs. II. NES, normalized enrichment score; *padj<0.05; **padj<0.01; *** *padj*<0.001. **(C, D, F, H, J-N)** Dot plots showing indicated gene expression within GABAergic neuron Subtype I and II. See also Table S8.

We further explored the differential characteristics between Subtype I and II (Table S8). Transcriptomic comparison of Subtypes I and II revealed 492 DEGs (Figure 9A). GSEA highlighted Subtype II's enrichment in neural development and functional pathways (Figure 9B-D), aligning with GO term analysis (Figure S9C-D) and pseudotime developmental trajectories (Figure 8D, 8E and S9F-H). In contrast, Subtype I exhibited elevated expression of ECM components (e.g., collagens), vesicle-related genes, and ER-linked translation activity (Figure 9E-H). Its top enriched KEGG pathways included ribosome biogenesis and ER protein processing (Figure 9I-K), alongside oxidative phosphorylation—features consistent with its immature state, where neurite formation pathways (motor proteins, and Hippo signaling) are less prioritized (Figure 9L-N).

Together, these findings demonstrate subtype-specific adaptive mechanisms to hypoxia-reoxygenation in GABAergic neurons, linking developmental maturation to hypoxia resilience (Subtype II) versus vulnerability (Subtype I), with Subtype III representing a transient stress response.

### Gene regulatory network alterations of various cell types in response to hypoxia-reoxygenation within vhCOs

**Figure 10:**
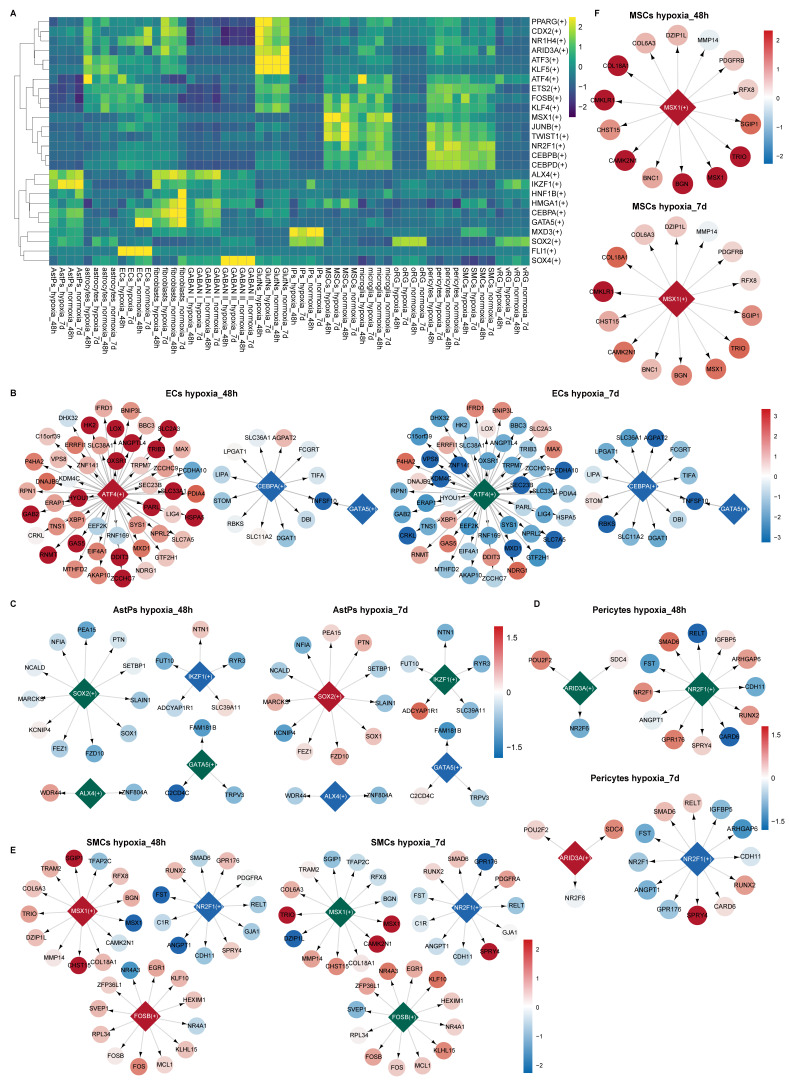
** SCENIC prediction of TF regulatory network alterations in each cell type within vhCOs during hypoxia-reoxygenation. (A)** Heatmap showing RSS and Z score of regulatory TFs in indicated cell types under hypoxic (hypoxia_48h), reoxygenated (hypoxia_7d) and normoxic (normoxia_48h/7d) conditions. GABAN I, GABAergic neurons Subtype I; GABAN II, GABAergic neurons Subtype II. See also Figure S11A. **(B-F)** Gene regulatory networks of selected top-ranked TFs in specific cell types under hypoxic (hypoxia_48h) and reoxygenated (hypoxia_7d) conditions. Color-coded diamonds denote upregulated (red), unchanged (green), and downregulated (blue) TF activities compared to normoxic vhCOs. Arrows indicate TF target genes, with expression levels color-coded by fold change (hypoxia_48h vs. normoxia_48h; hypoxia_7d vs. normoxia_7d), red and blue circles representing upregulation and downregulation respectively. See also Figure S10 and S11.

To delineate hypoxia-reoxygenation-induced transcriptional regulatory shifts in vhCOs, we applied SCENIC analysis, reconstructing cell type-specific regulon networks under different conditions. While core lineage regulators (e.g., *KLF5* in astrocytes, *FLI1* in ECs) remained stable across conditions, stress-responsive and cell-specific function-related transcription factors (TFs) exhibited dynamic activity (Figure 10A, S10A-O). Specifically, ECs, GABAN subtype I and microglia displayed upregulation of ATF4-driven UPR genes at 48-h post-hypoxia, normalizing after 5-day reoxygenation (Figure 10B and S11A-B). Additionally, ECs and GABAN subtype I suppressed the activity of *CEBPA*, a regulator of neural migration 45 and BBB integrity 46, and *GATA5*, an angiogenetic factor 47 (Figure 10B and S11A); whereas microglia enhanced the activity of *ARID3A*, perhaps involved in microglia development 48, and its target gene *SDC4* expression (Figure S11B). In AstPs, transient *IKZF1* downregulation at 48-h post-hypoxia preceded reoxygenation-phase suppression of *ALX4/GATA5* and activation of *SOX2* targets (Figure 10C).

As to BBB components, pericytes upregulated* ARID3A* and downregulated *NR2F1* activities at 5-day post-reoxygenation (Figure 10D); SMCs activated *MSX1*/*FOSB* that promotes ECM production 49 during 48-h hypoxia but sustained *NR2F1* suppression throughout hypoxia-reoxygenation (Figure 10E). Aligning with pseudotime and GSEA trends (Figure 6D and 6G), MSCs upregulated *MSX1* activity (Figure 10F) which promotes SMC differentiation 50. Notably, Fibroblasts suppressed fibrosis-linked regulators (*GATA5/CEBPA/HMGA1*) 51, 52 but activated *ARID3A/CDX2/PPARG* post-reoxygenation, the latter driving lipogenic pathways 53 (Figure S11C).

Collectively, these dynamic regulon alterations underscore hypoxia-reoxygenation-driven rewiring of TF networks that regulate stress adaptation with lineage-specific functional modulation.

## Discussion and Conclusion

In summary, leveraging single-cell transcriptomics in vhCOs modeling the fetal brain, our study delineates cell-type-specific responses and rewired intercellular communication networks underlying hypoxia-reoxygenation injury, while pinpointing vulnerable populations within neural and non-neural lineages. These findings provide mechanistic insights into cellular pathophysiology and potential therapeutic targets for fetal hypoxic brain injury.

Our study represents the first to analyze hypoxic fetal brain injury using vhCOs at single-cell resolution. By incorporating BBB elements alongside microglia, vhCOs overcome limitations of non-vascularized models 11-13, 54, enabling unprecedented investigation of neurovascular development and biphasic neuroinflammation. This human-centric platform resolves interspecies discrepancies inherent in animal studies. Pseudo-bulk GSEA of scRNA-seq data confirmed global transient activation of canonical hypoxia pathways during acute hypoxia and delayed inflammatory signaling post-reoxygenation, consistent with prior reports 7. However, cell-type-specific analysis revealed a critical divergence: most neural cells (excluding GABAergic neurons) exhibited transient hypoxic responses, whereas non-neural populations (astrocytes, microglia, pericytes, fibroblasts) sustained hypoxia signaling and glycolytic metabolism post-reoxygenation—a novel finding undetectable via bulk RNA-seq or immunostaining. Although hypoxia and reoxygenation are broadly linked to UPR activation 55, 56, SCENIC and GSEA analyses identified *ATF4*-driven UPR activation exclusively in ECs, GABAergic neuron Subtype I, and microglia during hypoxia While the ATF4 pathway is known to regulate TNF-α and IL-6 production in hypoxic mouse microglia 57, our vhCO model uncovered a striking divergence: microglia displayed biphasic inflammation—suppressed during hypoxia but hyperactivated post-reoxygenation—positioning them as a dual-edged sword: protective in acute stress but detrimental during recovery. This contrasts with animal models, where microglial pro-inflammatory activation (e.g., *AXL*, *CXCL1*, *NF-κB*) occurs during hypoxia 58-61. These discrepancies underscore the unique utility of vhCOs in modeling human-specific pathophysiology. And the lineage-specific adaptive mechanisms underscore the need to explore their functional consequences in neurovascular injury.

One important finding of our study is the identification of vulnerable neural-lineage cells (AstPs and GABAergic neuron Subtype I) within vhCOs exposed to hypoxia-reoxygenation. Unlike animal models of fetal hypoxic brain injury, which lack astrogliogenesis and thus cannot interrogate AstPs 7, our vhCOs revealed persistent developmental arrest and functional impairment in AstPs, marked by neurodegenerative transcriptional profiles. Notably, *CellPhoneDB* analysis detected upregulated IGF2-IGF2R signaling, a predicted neuroprotective interaction 62, from ECs to AstPs post-reoxygenation, suggesting a potential compensatory mechanism; however, its role in rescuing AstPs dysfunction requires longer-term observation. Additionally, whether targeting SOX2 activation post-reoxygenation in AstPs (revealed by SCENIC) can mitigate their impairment deserves investigation. These insights also extend to GABAergic neurons. While animal studies highlight hypoxia-induced gray matter injury, delayed interneuron maturation 7, and impaired GABAergic projection development 43, 44, the heterogeneity of these neurons has been largely overlooked. Our single-cell resolution approach resolved this gap, identifying immature GABAergic neurons (Subtype I; defined by low *GAD1/2* expression) as uniquely vulnerable to hypoxia-reoxygenation. These cells exhibited sustained suppression of developmental and synaptic genes, alongside neurodegenerative transcriptional profiles under hypoxia-reoxygenation. Although UPR activation is known to drive GABAergic deficits 63, whether *ATF4*-mediated UPR upregulation during hypoxia observed in GABAergic neurons Subtype I within vhCOs directly impairs their developmental status remains unclear, necessitating mechanistic exploration.

Another key finding of our study is the coordinated compartmentalized vasculature remodeling in non-neural cells (astrocytes, microglia, ECs, pericytes, SMCs, MSCs, fibroblasts) though hypoxia-reoxygenation. Early hypoxia suppressed endothelial angiogenic signaling (*EFNB2*, *MMP14*), destabilizing BBB through downregulation of tight junction proteins (*GJA1*, *JUP*, *TJP1*) in ECs. Concurrently, pericytes and SMCs initiated collagen deposition (*COL3A1*, *COL5A1*), which can reinforce the basement membrane 64. Post-reoxygenation, MSCs exhibited elevated collagen secretion and accelerated myogenic differentiation, while Notch signaling (*DLK1-NOTCH2*) from microglia, pericytes, SMCs, and fibroblasts drove vascular maturation, suggesting a self-repair mechanism aligning with murine studies 65. However, MSC depletion and fibroblast attrition may limit long-term vascular resilience, echoing findings in preterm infants with chronic cerebrovascular insufficiency 66. Notably, fibroblasts upregulated *ARID3A/CDX2/PPARG* post-reoxygenation, with *PPARG* driving lipogenic pathways 53, proving a potential therapeutic target that requires validation. Neural lineages also contributed to vascular recovery: neural progenitors facilitated BBB repair via *LGALS3-MERTK* signaling 42. These interconnected networks—spanning neurovascular crosstalk and metabolic reprogramming—reveal novel targets for enhancing hypoxia-reoxygenation tolerance.

While our vhCO model advances fetal hypoxia research, certain limitations remain. Notably, while BBB-associated markers are identified, functional validation of barrier integrity—such as permeability or P-glycoprotein activity—is limited by two key technical hurdles: (1) Matrigel's nonspecific adsorption of fluorescent tracers (e.g., dextran and peptides), which elevates background noise and confounds permeability assays (data not shown), and (2) the difficulty of achieving precise electrode placement at the BBB-like interface for reliable transendothelial electrical resistance measurements. Additionally, the absence of systemic circulation and peripheral immune cells restricts investigations into leukocyte infiltration and nutrient exchange dynamics. Furthermore, the model lacks sufficient oligodendrocytes and their precursors—a key population vulnerable to fetal hypoxia 7—limiting insights into their hypoxia-reoxygenation responses. Integrating microfluidic systems (e.g., microbead-based platforms) for dynamic BBB testing and improved nutrient delivery, alongside evaluating therapeutic interventions (e.g., anti-inflammatory agents) over prolonged culture periods, will further enhance the model's physiological relevance.

Crucially, reliance on PSCs from a single donor (i.e., H9) represents a significant constraint. While this enhances internal consistency for identifying cell type-specific responses within this genetic background, it fundamentally limits the generalizability of our findings across genetically diverse populations and crucially hinders our ability to distinguish between conserved, cell-intrinsic responses and potential donor-specific effects. Therefore, the observed phenotypes may reflect characteristics specific to the H9 line rather than universal responses. To address these gaps, future studies must prioritize validating key findings across multi-donor cohorts to disentangle conserved mechanisms from donor-specific effects and establish broader applicability.

Taken together, our study addresses key limitations in modeling human fetal brain injury by uncovering lineage-specific vulnerabilities and adaptive mechanisms unattainable in animal models and traditional non-vascularized organoids. The human-centric vhCO platform bridges translational gaps, providing a robust tool to develop potential therapeutics. By mapping cellular pathophysiology and intercellular networks, our study lays the foundation for precision medicine approaches to mitigate fetal hypoxic brain injury and its lifelong consequences.

## Supplementary Material

Supplementary figures, table 1, and table legends.

Supplementary table 2.

Supplementary table 3.

Supplementary table 4.

Supplementary table 5.

Supplementary table 6.

Supplementary table 7.

Supplementary table 8.

## Abbreviations

AstPs: Astrocyte precursors
BBB: Blood-brain barrier
DEGs: Differentially expressed genes
ECs: Endothelial cells
ECM: Extracellular matrix
ER: Endoplasmic reticulum
GABAN: GABAergic neurons
GO: Gene Ontology
GSEA: Gene Set Enrichment Analysis
hBVOs: Human blood vessel organoids
hCOs: Human cortical organoids
hESCs: Human embryonic stem cells
hPSCs: Human pluripotent stem cells
HIF-1α: Hypoxia-inducible factor 1-alpha
IGF2: Insulin-like growth factor 2
IGF2R: Insulin-like growth factor 2 receptor
IPs: Intermediate progenitors
KEGG: Kyoto Encyclopedia of Genes and Genomes
LGALS3: Galectin-3
MERTK: MER proto-oncogene tyrosine kinase
MSCs: Mesenchymal stromal cells
NES: Normalized enrichment score
NF-κB: Nuclear factor kappa-light-chain-enhancer of activated B cells
oRG: Outer radial glia
padj: Adjusted p-value
pcw: Post-conceptional weeks
RT-qPCR: Reverse transcription quantitative PCR
RG: Radial glia
SCENIC: Single-Cell Regulatory Network Inference and Clustering
scRNA-seq: Single-cell RNA-sequencing
SFRP2: Secreted frizzled-related protein 2
SMCs: Smooth muscle cells
SRCC: Spearman's rank correlation coefficient
TFs: Transcription factors
UPR: Unfolded protein response
UMAP: Uniform Manifold Approximation and Projection
vhCOs: Vascularized human cortical organoids
vRG: Ventricular radial glia
Wnts: Wingless-related integration site proteins
