# Supplementary material for: Single-cell transcriptomics of vascularized human brain organoids decipher lineage-specific stress adaptation in fetal hypoxia-reoxygenation injury
Source: Theranostics. 2025 Jun 9;15(14):7001–24. doi: 10.7150/thno.117001 (PMC12203816; doi:10.7150/thno.117001)
Supplement: Supplementary file 1 — Supplementary figures, table 1, and table legends. [file thnov15p7001s1.pdf]

Supplementary information

Supplementary figures

Supplementary figure legend

Figure S1 Characterization of hCOs and hBVOs induction from hESCs, related to Figure 1.

(A) Schematic diagram illustrating the differentiation process of hESCs into hCOs.

(B) Representative bright-field images of hCOs at distinct developmental stages, highlighting morphological maturation over time. Scale bars: 200 μm.

(C) Quantitative analysis of hCO diameter across different time points. Data are presented as mean ± SEM (n = 6). Statistical significance between groups was determined by one-way ANOVA test (*p < 0.05, **p < 0.01, ***p < 0.001).

(D) Schematic diagram depicting the stepwise differentiation protocol of hESCs into hBVOs.

(E) Bright-field images showing morphological progression of BVOs at key developmental time points, demonstrating structural maturation. Scale bars: 200 μm.

(F) Quantitative measurement of organoid diameter across sequential culture stages. Data are presented as mean ± SD (n = 5 organoids per group). Statistical significance was analyzed by one-way ANOVA test (*p < 0.05, **p < 0.01, ***p < 0.001).

Figure S2. Characterization of single-cell transcriptome in vhCOs, related to Figure 3.

(A-C) Violin plots displaying the distributions of (A) total read counts, (B) detected genes per cell (C) mitochondrial gene content across each sample after quality control.

(D-H) Transcriptional analyses of normoxic, hypoxic and reoxygenated vhCOs, mapping onto the developing and adult human brain (age 4 pcw to >60 years) using Spearman's rank correlation coefficient. Related to Figure 3D. pcw, post-conceptional weeks; mos, months; yrs, years. CGE, caudal ganglionic eminence; MGE, medial ganglionic eminence; HIP, hippocampus; M1C-S1C, primary motor-sensory cortex (samples); MFC, anterior cingulate (medial prefrontal) cortex; Ocx, occipital neocortex; OFC, orbital frontal cortex; PCx, parietal neocortex; STC, posterior (caudal) superior temporal cortex; VFC, ventrolateral prefrontal cortex.

Figure S3. Cell type annotation of single-cell transcriptome in vhCOs, related to Figure 3.

(A) UMAP visualization of the single-cell transcriptome of vhCOs (n=46,418 cells), colored by clusters. Related to Figure 3E-3F.

(B) Violin plots displaying cell type-specific gene expression to each cell cluster. oRG, outer radial glia; vRG, ventricular radial glia, related to Figure 3G.

Figure S4. Feature plots showing cell-type-specific marker expression in vhCOs, related to Figure 3G.

Figure S5. Gene ontology enrichment analysis of DEGs across the 16 cell clusters in vhCOs, related to Figure 3H.

oRG, outer radial glia; vRG, ventricular radial glia.

Figure S6. Transcriptional analyses of each cell type in normoxic, hypoxic and reoxygenated vhCOs, mapping onto the developing and adult human brain, related to Figure 3D.

pcw, post-conceptional weeks; mos, months; yrs, years; oRG, outer radial glia; vRG, ventricular radial glia.

Figure S7. Transcriptional characteristics of each cell type in normoxic, hypoxic and reoxygenated vhCOs, related to Figure 5 and 6.

(A and B) The number of DEGs in indicated (A) neural-lineage and non-neural lineage (B) cell type of hypoxic and reoxygenated vhCOs compared to that of normoxic vhCOs. GABANs, GABAergic neurons; AstPs, astrocyte precursors; GlutNs, glutamatergic neurons; IPs, intermediate progenitors; oRG, outer radial glia; vRG, ventricular radial glia; ECs, endothelial cells; MSCs, mesenchymal stromal cells; SMCs, smooth muscle cells.

(C) Bar plots showing neurodegenerative disease-associated genes expression within indicated neural-lineage cell types of hypoxic and reoxygenated vhCOs compared to that of normoxic vhCOs, related to Figure 5A.

Figure S8. Ligand-receptor pairs prediction of each cell type in vhCOs during hypoxia-reoxygenation, related to Figure 7.

(A) CellChat-generated network plots visualizing ligand-receptor interactions (color-coded) between each indicated cell type in vhCOs under normoxic (normoxia_48h/7d), hypoxic (hypoxia_48h), and reoxygenated (hypoxia_7d) conditions. Node size scales with interaction strength, while line thickness reflects communication probability.

(B) CellChat-predicted top 10 ligand-receptor interactions between neural lineages in vhCOs under normoxic, hypoxic (hypoxia_48h), and reoxygenated (hypoxia_7d) conditions, visualized with sizes and colors corresponding to cell-cell communication (CCC) scores.

Figure S9. Characteristics of GABAergic neuron subtypes during hypoxia-reoxygenation, related to Figure 8.

(A) Violin plots displaying subtype-specific gene expression to each cell cluster, related to Figure 8A-8C.

(B) Feature plots displaying subtype-specific gene expression within GABAergic neurons, related to Figure 8A-8C.

(C-E) Gene ontology enrichment analysis of DEGs across the 3 subtypes within GABAergic neurons, related to Figure 8A-8C.

(F-H) Heatmap displaying the expression level of TOP 100 DEGs in each branch with pseudo- time, related to Figure 8D-8E.

Figure S10. SCENIC analyses of each cell type during hypoxia-reoxygenation within vhCOs, related to Figure 10.

(A) Dot plots displaying RSS and Z score of transcription factors (TFs) within each indicated cell type in normoxic (normoxia_48h/7d), hypoxic (hypoxia_48h), and reoxygenated (hypoxia_7d) vhCOs, related to Figure 10A. RSS, regulon specificity scores. GABAN I, GABAergic neurons Subtype I; GABAN II, GABAergic neurons Subtype II.

(B-O) SCENIC plots displaying RSS of top 5 TFs within each indicated cell type in normoxic (normoxia_48h/7d), hypoxic (hypoxia_48h), and reoxygenated (hypoxia_7d) vhCOs. GABAN, GABAergic neurons.

Figure S11.TF regulatory network alterations during hypoxia-reoxygenation within vhCOs, related to Figure 10.

(A-C) TF regulatory networks of specific cell types in hypoxic (hypoxia_48h) and

reoxygenated (hypoxia_7d) vhCOs. Color-coded diamonds denote upregulated (red), unchanged (green), and downregulated (blue) TF activities compared to normoxic (normoxia_48h/7d) vhCOs. Arrows indicate TF target genes, with expression levels color-coded by fold change (hypoxia_48h vs. normoxia_48h; hypoxia_7d vs. normoxia_7d), red and blue circles representing upregulation and downregulation respectively.

Supplementary Tables
Table S1. Key resources.
Table S2. GO enrichment of each cell type.
Table S3. pseudo-bulk gene expression and GSEA (vs normoxia).
Table S4. DEG of each cell type (hypoxia vs normoxia).
Table S5. GSEA of each cell type (hypoxia vs normoxia).
Table S6. GO enrichment of each GABAergic neuron subtype.
Table S7. GABAergic neuron subtype DEG & GSEA (vs normoxia).
Table S8. DEG and GSEA of GABAergic neuron subtype I vs II.

Table S1. Key resources.

**Antibodies**				
**Name**	**Application / Dilution**	**Source**	**RRID**	**Cat#**
anti-SOX1	IF (1:100)	Bio-Techne	AB_2239879	AF3369-SP
anti-PAX6	IF (1:350)	Abcam	AB_2750924	AB195045
anti-MAP2	IF (1:500)	Abcam	AB_2895301	AB183830
anti-SOX2	IF (1:350)	Abcam	AB_10710406	AB79351
Anti-TBR1	IF (1:100)	Abcam	AB_2936859	AB183032
Anti-TBR2	IF (1:100)	Abcam	AB_778267	AB23345
Anti-HIF-1α	IF (1:100)	Cell Signaling Technology	AB_2799095	36169T
Anti-CD31 (mouse)	IF (1:200)	Abcam	AB_307284	AB9498
Anti-CD31 (goat)	IF (1:100)	R&D systems	AB_2161028	AF3628-SP
Anti-ACTA2	IF (1:200)	Abclonal	AB_2861755	A17910
Anti-PDGFRβ	IF (1:100)	Bio-Techne	AB_355339A	F385-SP
Anti-GFAP	IF (1:100)	Abclonal	AB_2757050	A0237
Anti-S100B	IF (1:100)	Proteintech	AB_2254244	15146-1-AP
Anti-CLDN5	IF (1:100)	Santa Cruz Biotechnology	AB_10988234	sc-374221
Anti-ZO-1	IF (1:100)	Abclonal	-	A0659
Alexa Fluor 488-Conjugated anti-mouse	IF (1:500)	Abcam	AB_2732856	AB150105
Alexa Fluor 594-conjugated anti-rabbit	IF (1:500)	Abcam	AB_2782993	AB150076
Alexa Fluor 647-conjugated anti-goat	IF (1:500)	Abcam	AB_2687955	AB150135

Figure S1

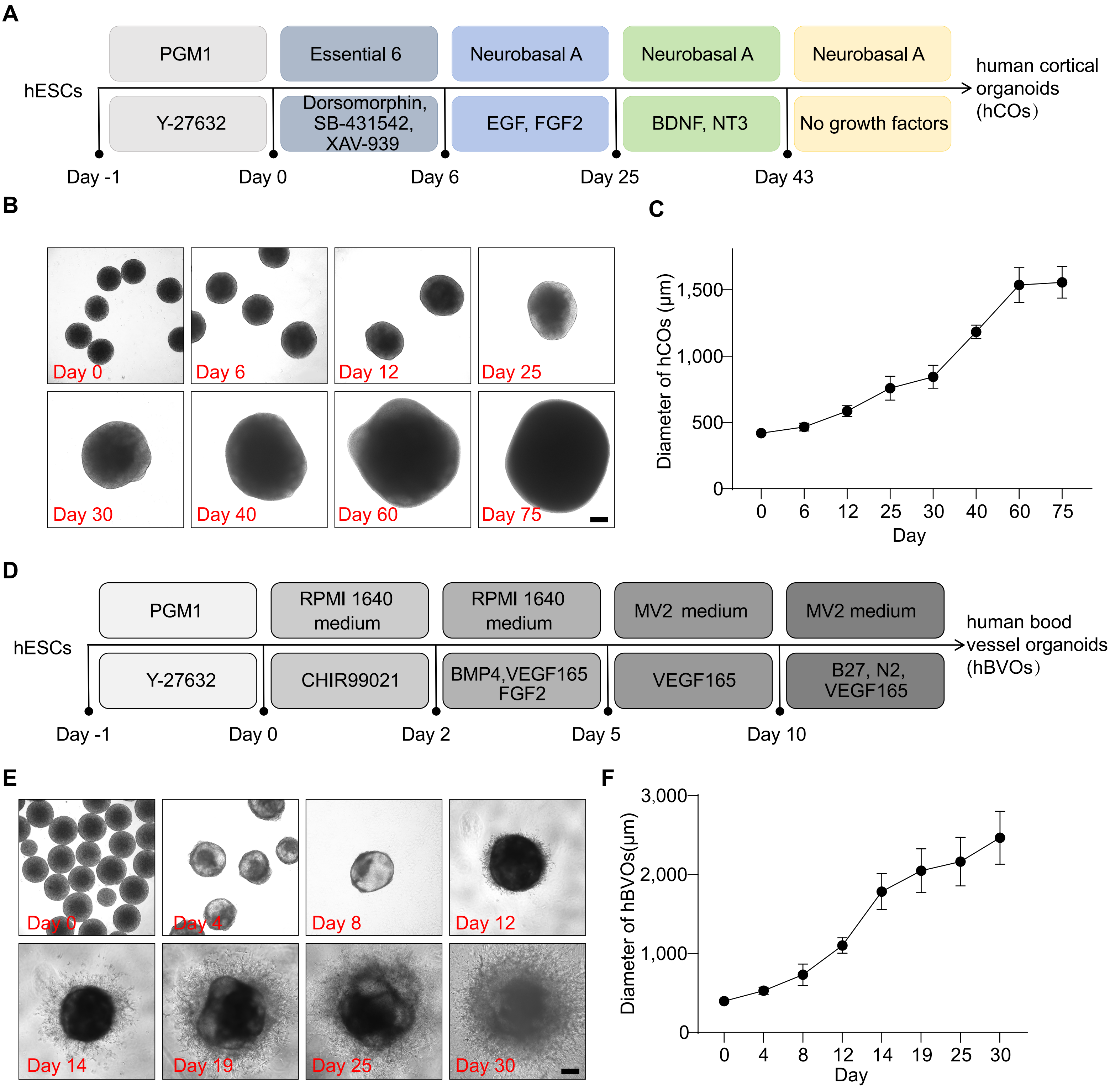
A
PGM1
Essential 6
Neurobasal A
Neurobasal A
Neurobasal A
hESCs
Y-27632
Dorsomorphin, SB-431542, XAV-939
EGF, FGF2
BDNF, NT3
No growth factors
human cortical organoids (hCOs)
Day -1
Day 0
Day 6
Day 25
Day 43
B
Day 0
Day 6
Day 12
Day 25
Day 30
Day 40
Day 60
Day 75
C
Diameter of hCOs (μm)
1,500
1,000
500
0
0
6
12
25
30
40
60
75
Day
D
PGM1
RPMI 1640 medium
RPMI 1640 medium
MV2 medium
MV2 medium
hESCs
Y-27632
CHIR99021
BMP4,VEGF165 FGF2
VEGF165
B27, N2, VEGF165
human bood vessel organoids (hBVOs)
Day -1
Day 0
Day 2
Day 5
Day 10
E
Day 0
Day 4
Day 8
Day 12
Day 14
Day 19
Day 25
Day 30
F
Diameter of hBVOs(μm)
3,000
2,000
1,000
0
0
4
8
12
14
19
25
30
Day

Figure S2

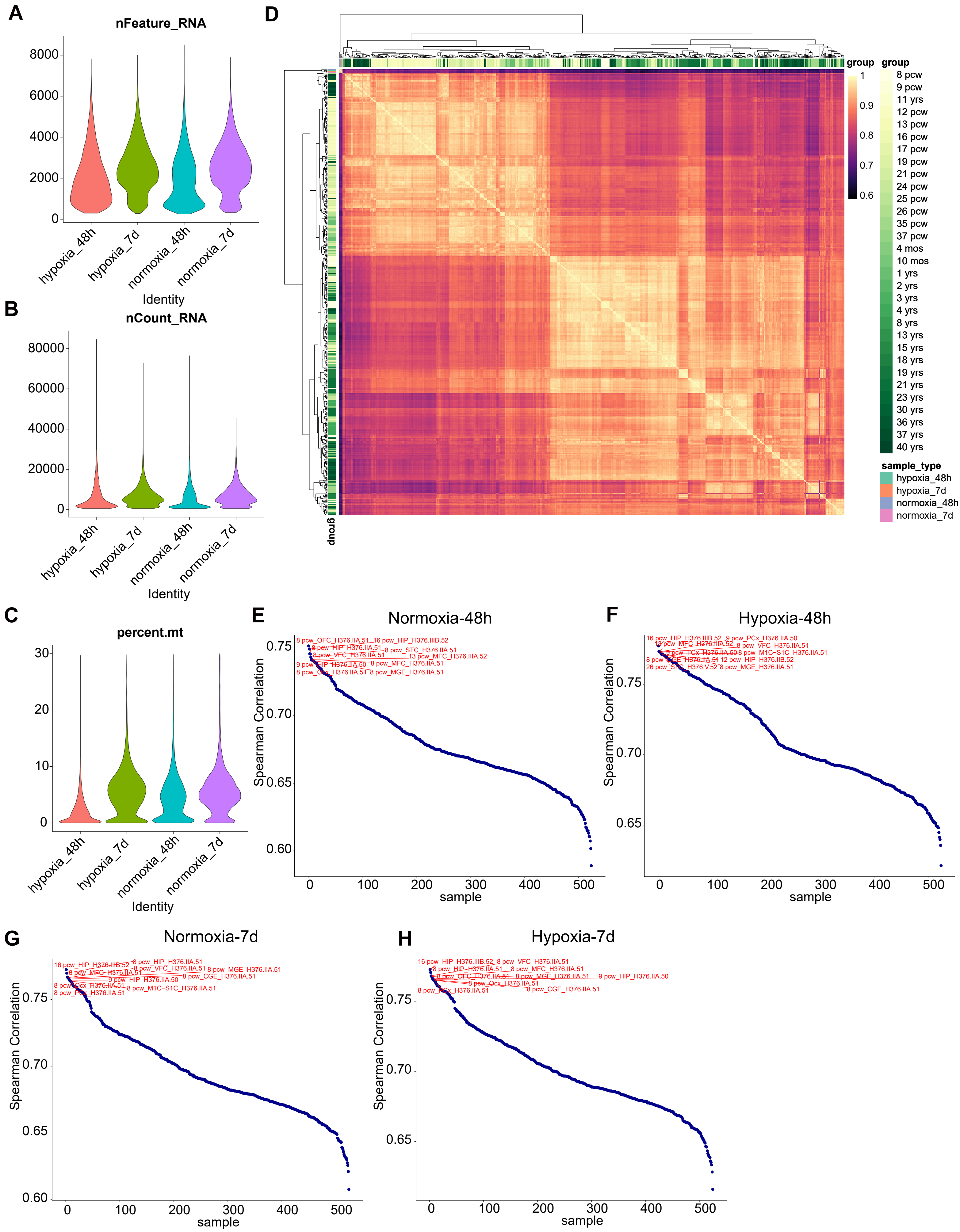

A
nFeature_RNA
0
2000
4000
6000
8000
hypoxia_48h
hypoxia_7d
normoxia_48h
normoxia_7d
Identity
B
nCount_RNA
0
20000
40000
60000
80000
hypoxia_48h
hypoxia_7d
normoxia_48h
normoxia_7d
Identity
C
percent.mt
0
10
20
30
hypoxia_48h
hypoxia_7d
normoxia_48h
normoxia_7d
Identity
D
group
group
1
0.9
0.8
0.7
0.6
8 pcw
9 pcw
11 yrs
12 pcw
13 pcw
16 pcw
17 pcw
19 pcw
21 pcw
24 pcw
25 pcw
26 pcw
35 pcw
37 pcw
4 mos
10 mos
1 yrs
2 yrs
3 yrs
4 yrs
8 yrs
13 yrs
15 yrs
18 yrs
19 yrs
21 yrs
23 yrs
30 yrs
36 yrs
37 yrs
40 yrs
sample_type
hypoxia_48h
hypoxia_7d
normoxia_48h
normoxia_7d
E
Normoxia-48h
Spearman Correlation
0.60
0.65
0.70
0.75
0
100
200
300
400
500
sample
8 pcw_OFC_H376.IIA.51
16 pcw_HIP_H376.IIIB.52
8 pcw_HIP_H376.IIA.51
8 pcw_STC_H376.IIA.51
8 pcw_VFC_H376.IIA.51
13 pcw_MFC_H376.IIIA.52
9 pcw_HIP_H376.IIA.50
8 pcw_MFC_H376.IIA.51
8 pcw_Ocx_H376.IIA.51
8 pcw_MGE_H376.IIA.51
F
Hypoxia-48h
Spearman Correlation
0.65
0.70
0.75
0
100
200
300
400
500
sample
16 pcw_HIP_H376.IIIB.52
9 pcw_PCx_H376.IIA.50
13 pcw_MFC_H376.IIIA.52
8 pcw_VFC_H376.IIA.51
9 pcw_TCx_H376.IIA.50
8 pcw_M1C-S1C_H376.IIA.51
8 pcw_CGE_H376.IIA.51
12 pcw_HIP_H376.IIB.52
26 pcw_STC_H376.V.52
8 pcw_MGE_H376.IIA.51
G
Normoxia-7d
Spearman Correlation
0.60
0.65
0.70
0.75
0
100
200
300
400
500
sample
16 pcw_HIP_H376.IIIB.52
8 pcw_HIP_H376.IIA.51
8 pcw_VFC_H376.IIA.51
8 pcw_MFC_H376.IIA.51
8 pcw_MGE_H376.IIA.51
8 pcw_CGE_H376.IIA.51
9 pcw_HIP_H376.IIA.50
8 pcw_Ocx_H376.IIA.51
8 pcw_M1C-S1C_H376.IIA.51
8 pcw_PCx_H376.IIA.51
H
Hypoxia-7d
Spearman Correlation
0.65
0.70
0.75
0
100
200
300
400
500
sample
16 pcw_HIP_H376.IIIB.52
8 pcw_VFC_H376.IIA.51
8 pcw_HIP_H376.IIA.51
8 pcw_MFC_H376.IIA.51
8 pcw_OFC_H376.IIA.51
8 pcw_MGE_H376.IIA.51
9 pcw_HIP_H376.IIA.50
8 pcw_Ocx_H376.IIA.51
8 pcw_PCx_H376.IIA.51
8 pcw_CGE_H376.IIA.51

Figure S3

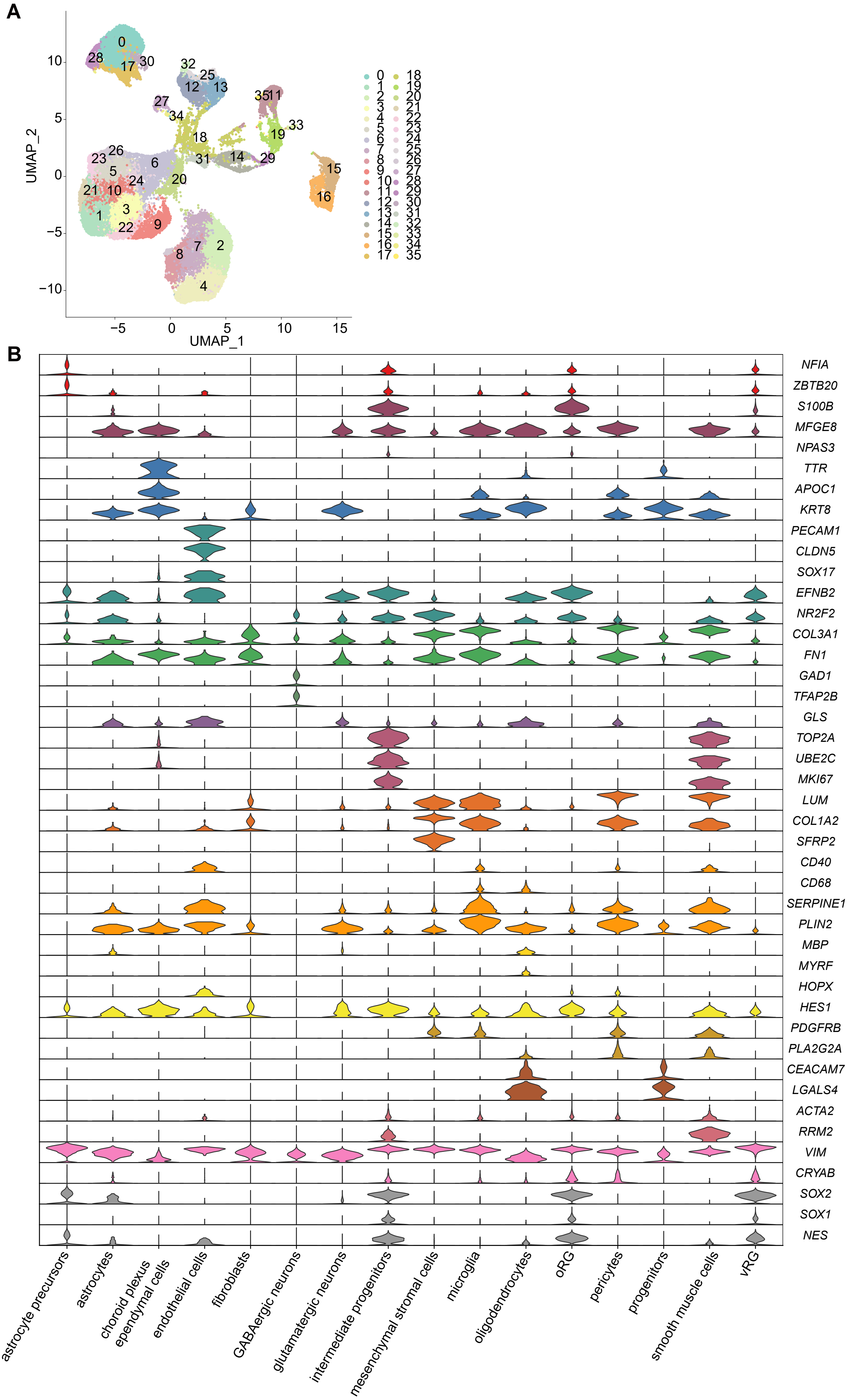
A
UMAP_2
UMAP_1
B
NFIA
ZBTB20
S100B
MFGE8
NPAS3
TTR
APOC1
KRT8
PECAM1
CLDN5
SOX17
EFNB2
NR2F2
COL3A1
FN1
GAD1
TFAP2B
GLS
TOP2A
UBE2C
MKI67
LUM
COL1A2
SFRP2
CD40
CD68
SERPINE1
PLIN2
MBP
MYRF
HOPX
HES1
PDGFRB
PLA2G2A
CEACAM7
LGALS4
ACTA2
RRM2
VIM
CRYAB
SOX2
SOX1
NES
astrocyte precursors
astrocytes
choroid plexus ependymal cells
endothelial cells
fibroblasts
GABAergic neurons
glutamatergic neurons
intermediate progenitors
mesenchymal stromal cells
microglia
oligodendrocytes
oRG
pericytes
progenitors
smooth muscle cells
vRG

Figure S4

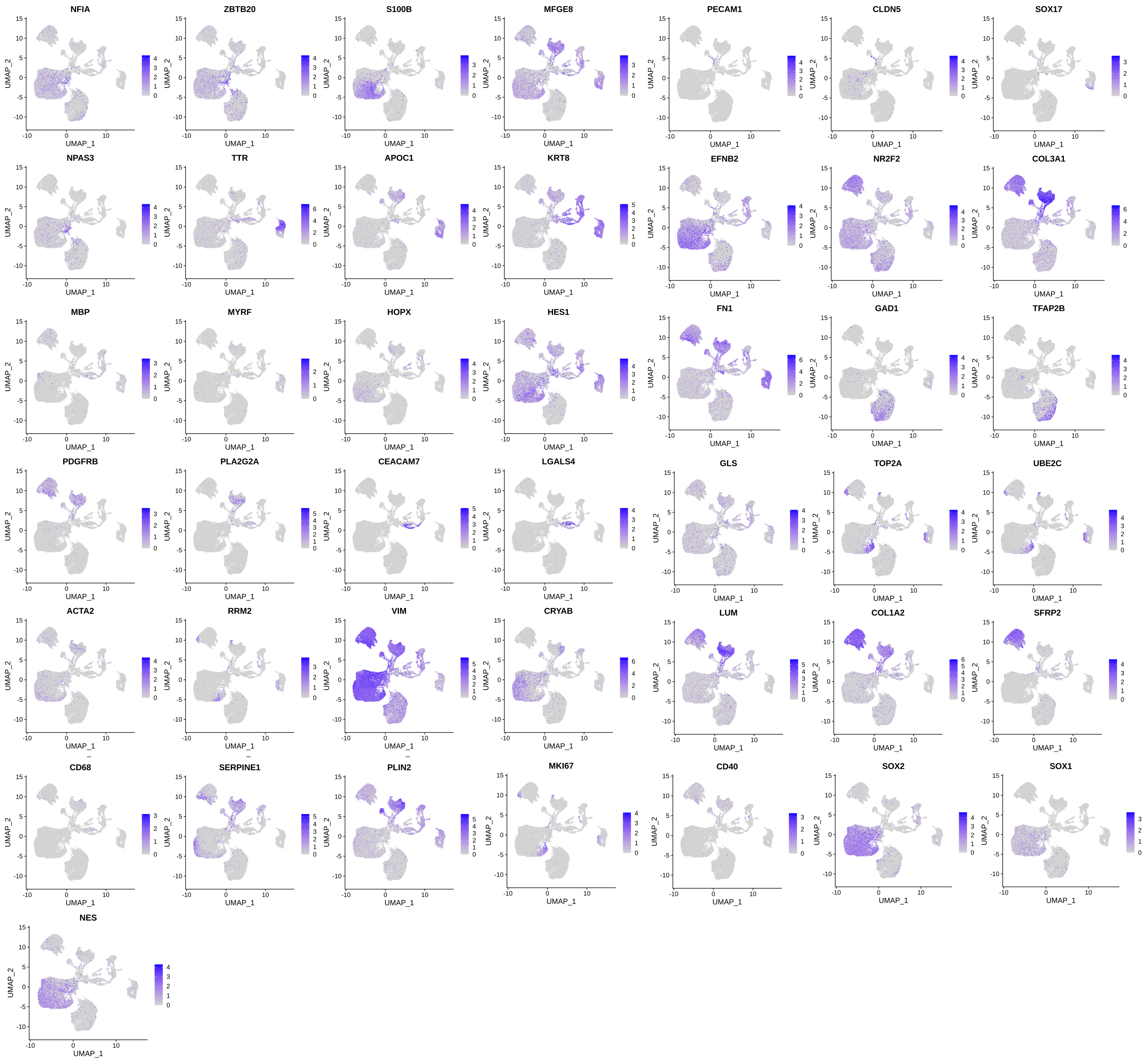
NFIA
ZBTB20
S100B
MFGE8
PECAM1
CLDN5
SOX17
NPAS3
TTR
APOC1
KRT8
EFNB2
NR2F2
COL3A1
MBP
MYRF
HOPX
HES1
FN1
GAD1
TFAP2B
PDGFRB
PLA2G2A
CEACAM7
LGALS4
GLS
TOP2A
UBE2C
ACTA2
RRM2
VIM
CRYAB
LUM
COL1A2
SFRP2
CD68
SERPINE1
PLIN2
MKI67
CD40
SOX2
SOX1
NES
UMAP_1
UMAP_2

Figure S5

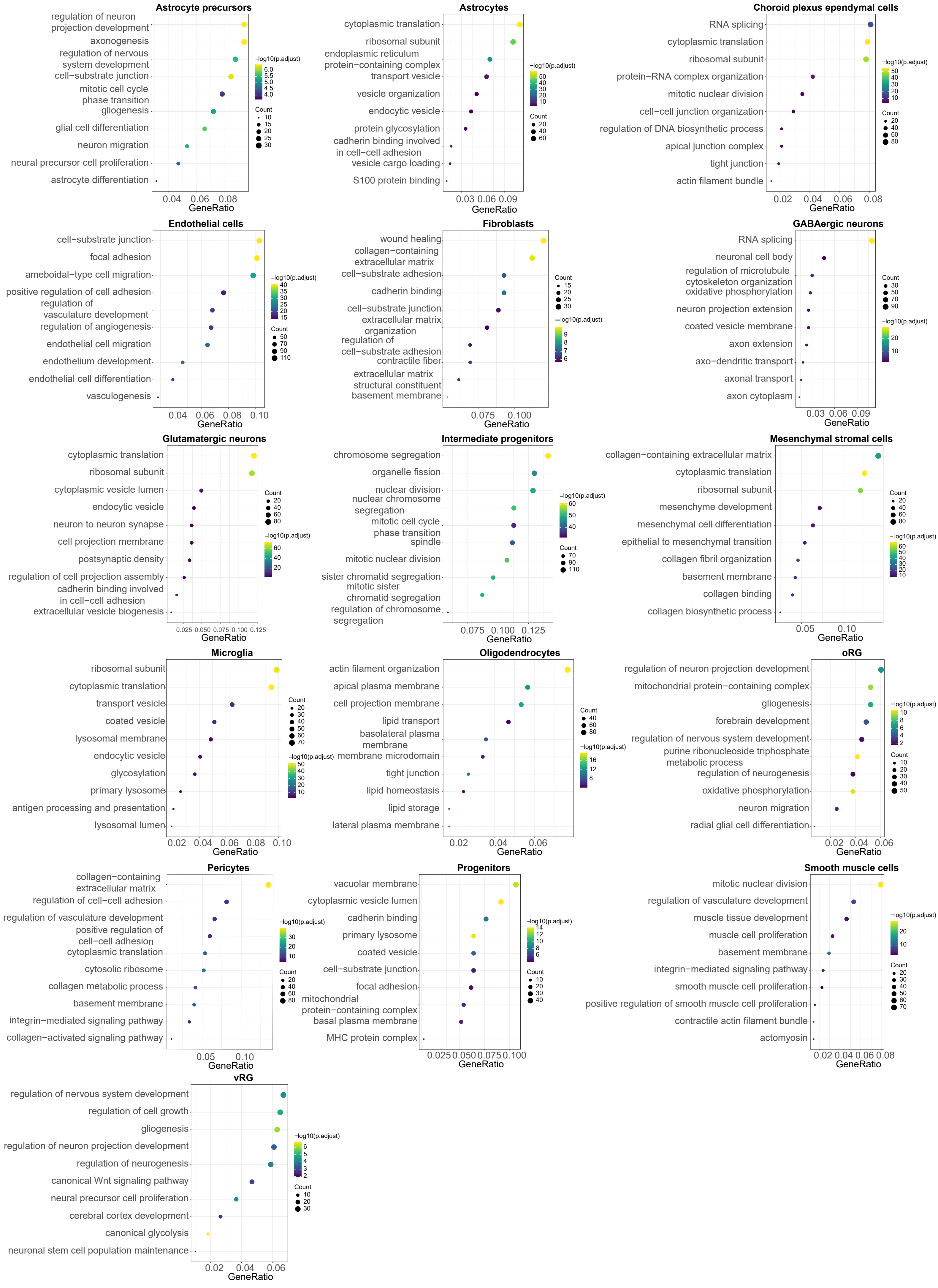

Astrocyte precursors
regulation of neuron projection development
axonogenesis
regulation of nervous system development
cell−substrate junction
mitotic cell cycle phase transition
gliogenesis
glial cell differentiation
neuron migration
neural precursor cell proliferation
astrocyte differentiation
GeneRatio
−log10(p.adjust)
Count
Astrocytes
cytoplasmic translation
ribosomal subunit
endoplasmic reticulum protein−containing complex
transport vesicle
vesicle organization
endocytic vesicle
protein glycosylation
cadherin binding involved in cell−cell adhesion
vesicle cargo loading
S100 protein binding
GeneRatio
Choroid plexus ependymal cells
RNA splicing
cytoplasmic translation
ribosomal subunit
protein−RNA complex organization
mitotic nuclear division
cell−cell junction organization
regulation of DNA biosynthetic process
apical junction complex
tight junction
actin filament bundle
GeneRatio
Endothelial cells
cell−substrate junction
focal adhesion
ameboidal−type cell migration
positive regulation of cell adhesion
regulation of vasculature development
regulation of angiogenesis
endothelial cell migration
endothelium development
endothelial cell differentiation
vasculogenesis
GeneRatio
Fibroblasts
wound healing
collagen−containing extracellular matrix
cell−substrate adhesion
cadherin binding
cell−substrate junction
extracellular matrix organization
regulation of cell−substrate adhesion
contractile fiber
extracellular matrix structural constituent
basement membrane
GeneRatio
GABAergic neurons
RNA splicing
neuronal cell body
regulation of microtubule cytoskeleton organization
oxidative phosphorylation
neuron projection extension
coated vesicle membrane
axon extension
axo−dendritic transport
axonal transport
axon cytoplasm
GeneRatio
Glutamatergic neurons
cytoplasmic translation
ribosomal subunit
cytoplasmic vesicle lumen
endocytic vesicle
neuron to neuron synapse
cell projection membrane
postsynaptic density
regulation of cell projection assembly
cadherin binding involved in cell−cell adhesion
extracellular vesicle biogenesis
GeneRatio
Intermediate progenitors
chromosome segregation
organelle fission
nuclear division
nuclear chromosome segregation
mitotic cell cycle phase transition
spindle
mitotic nuclear division
sister chromatid segregation
mitotic sister chromatid segregation
regulation of chromosome segregation
GeneRatio
Mesenchymal stromal cells
collagen−containing extracellular matrix
cytoplasmic translation
ribosomal subunit
mesenchyme development
mesenchymal cell differentiation
epithelial to mesenchymal transition
collagen fibril organization
basement membrane
collagen binding
collagen biosynthetic process
GeneRatio
Microglia
ribosomal subunit
cytoplasmic translation
transport vesicle
coated vesicle
lysosomal membrane
endocytic vesicle
glycosylation
primary lysosome
antigen processing and presentation
lysosomal lumen
GeneRatio
Oligodendrocytes
actin filament organization
apical plasma membrane
cell projection membrane
lipid transport
basolateral plasma membrane
membrane microdomain
tight junction
lipid homeostasis
lipid storage
lateral plasma membrane
GeneRatio
oRG
regulation of neuron projection development
mitochondrial protein−containing complex
gliogenesis
forebrain development
regulation of nervous system development
purine ribonucleoside triphosphate metabolic process
regulation of neurogenesis
oxidative phosphorylation
neuron migration
radial glial cell differentiation
GeneRatio
Pericytes
collagen−containing extracellular matrix
regulation of cell−cell adhesion
regulation of vasculature development
positive regulation of cell−cell adhesion
cytoplasmic translation
cytosolic ribosome
collagen metabolic process
basement membrane
integrin−mediated signaling pathway
collagen−activated signaling pathway
GeneRatio
Progenitors
vacuolar membrane
cytoplasmic vesicle lumen
cadherin binding
primary lysosome
coated vesicle
cell−substrate junction
focal adhesion
mitochondrial protein−containing complex
basal plasma membrane
MHC protein complex
GeneRatio
Smooth muscle cells
mitotic nuclear division
regulation of vasculature development
muscle tissue development
muscle cell proliferation
basement membrane
integrin−mediated signaling pathway
smooth muscle cell proliferation
positive regulation of smooth muscle cell proliferation
contractile actin filament bundle
actomyosin
GeneRatio
vRG
regulation of nervous system development
regulation of cell growth
gliogenesis
regulation of neuron projection development
regulation of neurogenesis
canonical Wnt signaling pathway
neural precursor cell proliferation
cerebral cortex development
canonical glycolysis
neuronal stem cell population maintenance
GeneRatio

Figure S6

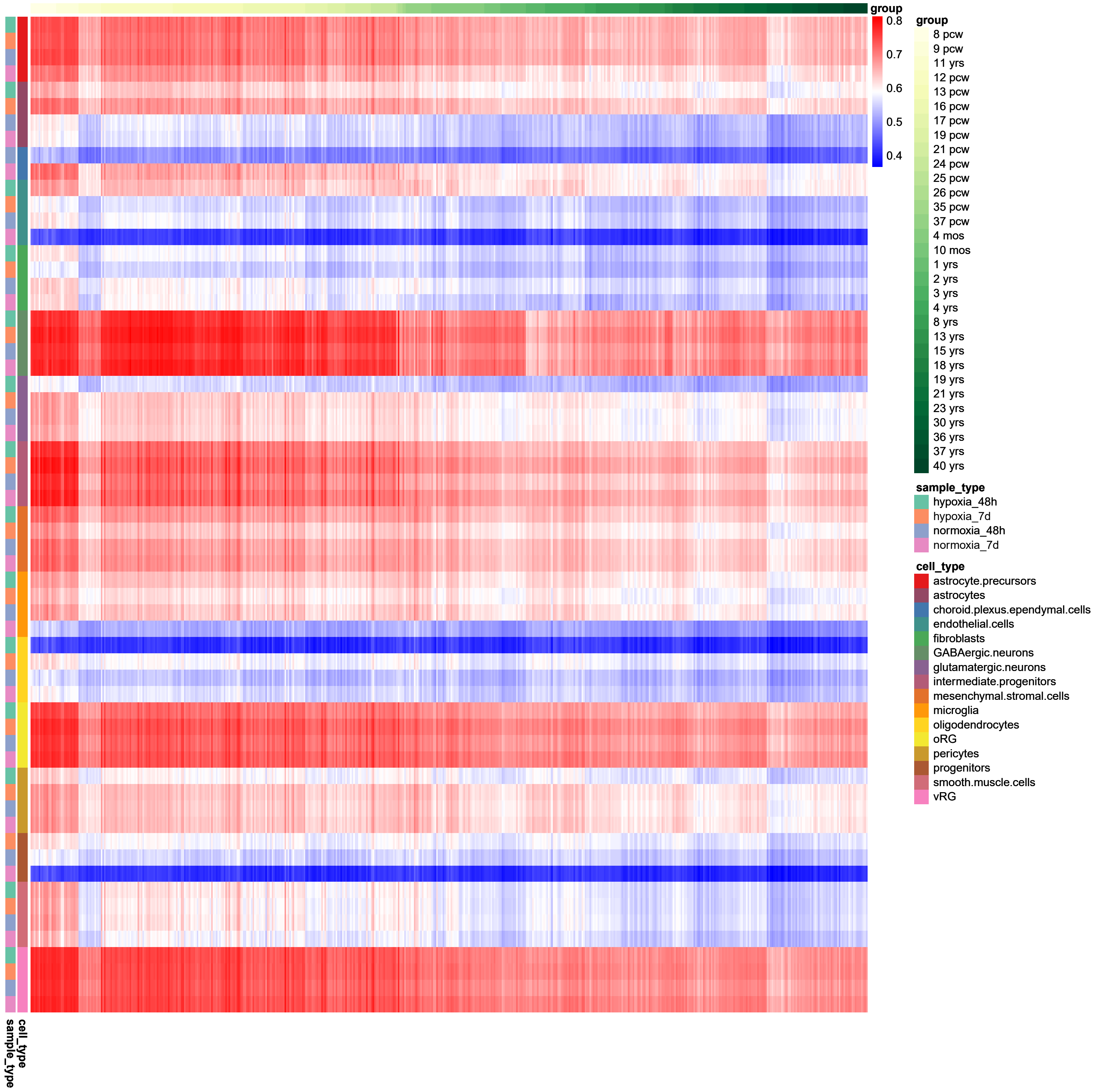

group
0.8
0.7
0.6
0.5
0.4
group
8 pcw
9 pcw
11 yrs
12 pcw
13 pcw
16 pcw
17 pcw
19 pcw
21 pcw
24 pcw
25 pcw
26 pcw
35 pcw
37 pcw
4 mos
10 mos
1 yrs
2 yrs
3 yrs
4 yrs
8 yrs
13 yrs
15 yrs
18 yrs
19 yrs
21 yrs
23 yrs
30 yrs
36 yrs
37 yrs
40 yrs
sample_type
hypoxia_48h
hypoxia_7d
normoxia_48h
normoxia_7d
cell_type
astrocyte.precursors
astrocytes
choroid.plexus.ependymal.cells
endothelial.cells
fibroblasts
GABAergic.neurons
glutamatergic.neurons
intermediate.progenitors
mesenchymal.stromal.cells
microglia
oligodendrocytes
oRG
pericytes
progenitors
smooth.muscle.cells
vRG
cell_type
sample_type

Figure S7

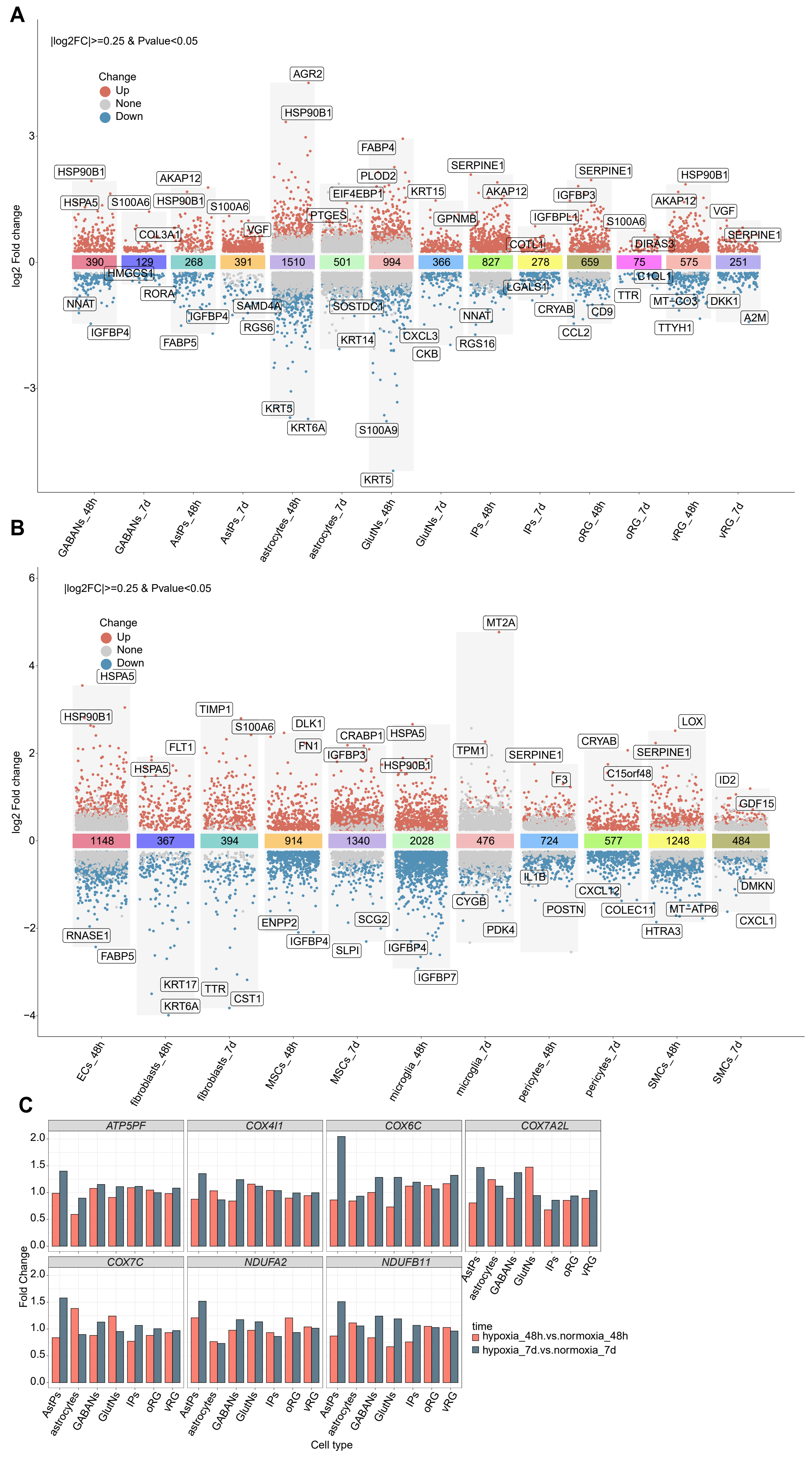
A
|log2FC|>=0.25 & Pvalue<0.05
Change
Up
None
Down
log2 Fold change
3
0
−3
AGR2
HSP90B1
FABP4
HSP90B1
AKAP12
PLOD2
SERPINE1
SERPINE1
HSP90B1
HSPA5
S100A6
HSP90B1
S100A6
EIF4EBP1
KRT15
AKAP12
IGFBP3
AKAP12
VGF
PTGES
GPNMB
IGFBPL1
S100A6
COL3A1
VGF
COTL1
DIRAS3
SERPINE1
390
129
268
391
1510
501
994
366
827
278
659
75
575
251
HMGCS1
C1QL1
NNAT
RORA
LGALS1
TTR
MT−CO3
DKK1
IGFBP4
SAMD4A
SOSTDC1
CRYAB
CD9
A2M
IGFBP4
RGS6
NNAT
CCL2
TTYH1
FABP5
KRT14
CXCL3
RGS16
CKB
KRT5
KRT6A
S100A9
KRT5
GABANs_48h
GABANs_7d
AstPs_48h
AstPs_7d
astrocytes_48h
astrocytes_7d
GlutNs_48h
GlutNs_7d
IPs_48h
IPs_7d
oRG_48h
oRG_7d
vRG_48h
vRG_7d
B
|log2FC|>=0.25 & Pvalue<0.05
Change
Up
None
Down
log2 Fold change
6
4
2
0
−2
−4
MT2A
HSPA5
HSP90B1
TIMP1
S100A6
DLK1
CRABP1
HSPA5
LOX
CRYAB
FLT1
FN1
IGFBP3
TPM1
SERPINE1
SERPINE1
HSPA5
HSP90B1
F3
C15orf48
ID2
GDF15
1148
367
394
914
1340
2028
476
724
577
1248
484
IL1B
DMKN
CXCL12
CYGB
POSTN
COLEC11
MT−ATP6
CXCL1
HTRA3
ENPP2
SCG2
PDK4
RNASE1
IGFBP4
SLPI
IGFBP4
FABP5
IGFBP7
KRT17
TTR
CST1
KRT6A
ECs_48h
fibroblasts_48h
fibroblasts_7d
MSCs_48h
MSCs_7d
microglia_48h
microglia_7d
pericytes_48h
pericytes_7d
SMCs_48h
SMCs_7d
C
ATP5PF
COX4I1
COX6C
COX7A2L
COX7C
NDUFA2
NDUFB11
Fold Change
2.0
1.5
1.0
0.5
0.0
AstPs
astrocytes
GABANs
GlutNs
IPs
oRG
vRG
time
hypoxia_48h.vs.normoxia_48h
hypoxia_7d.vs.normoxia_7d
Cell type

Figure S8

A

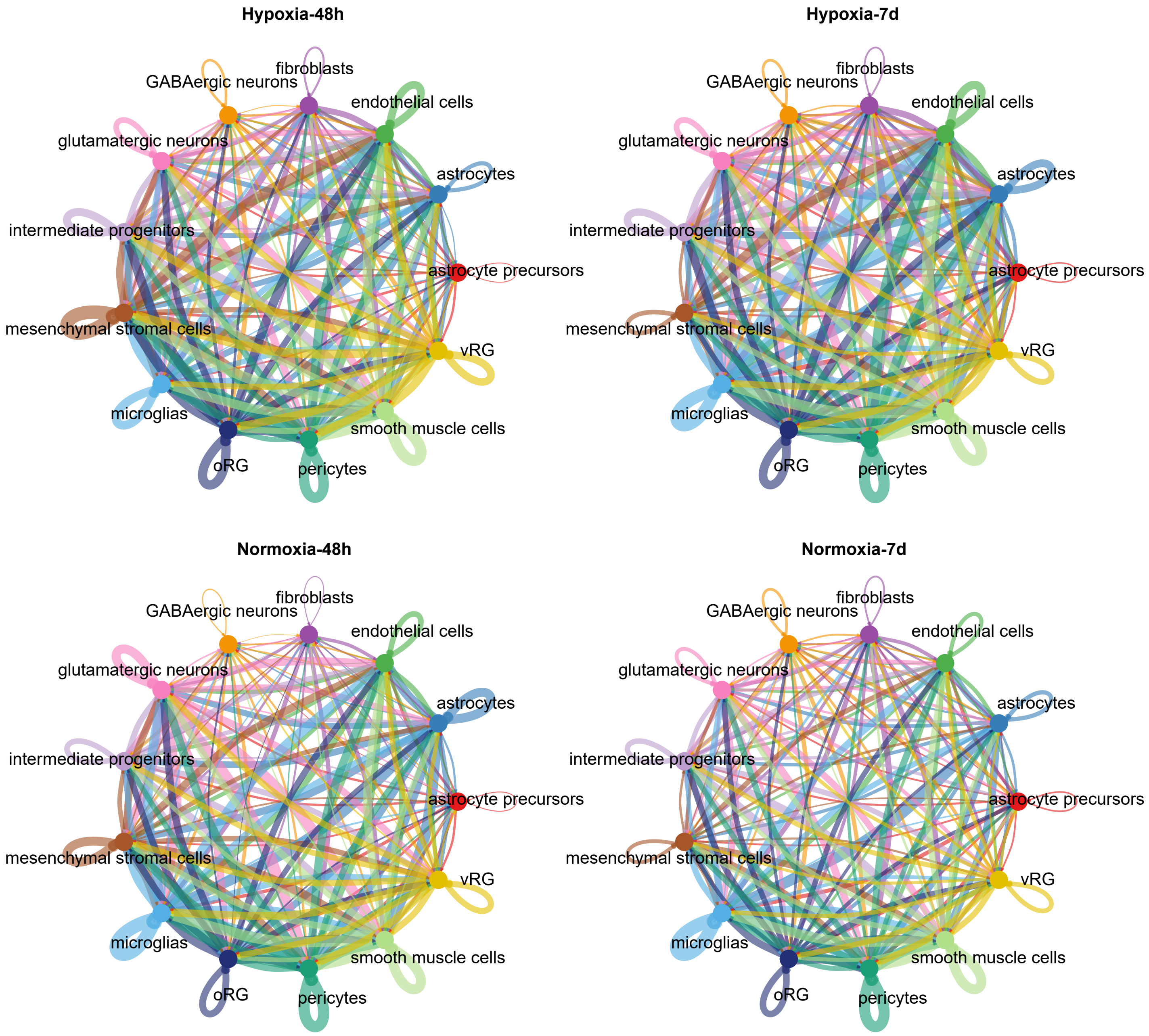

Hypoxia-48h
Hypoxia-7d
Normoxia-48h
Normoxia-7d
fibroblasts
GABAergic neurons
endothelial cells
glutamatergic neurons
astrocytes
intermediate progenitors
astrocyte precursors
mesenchymal stromal cells
vRG
microglias
smooth muscle cells
oRG
pericytes

B

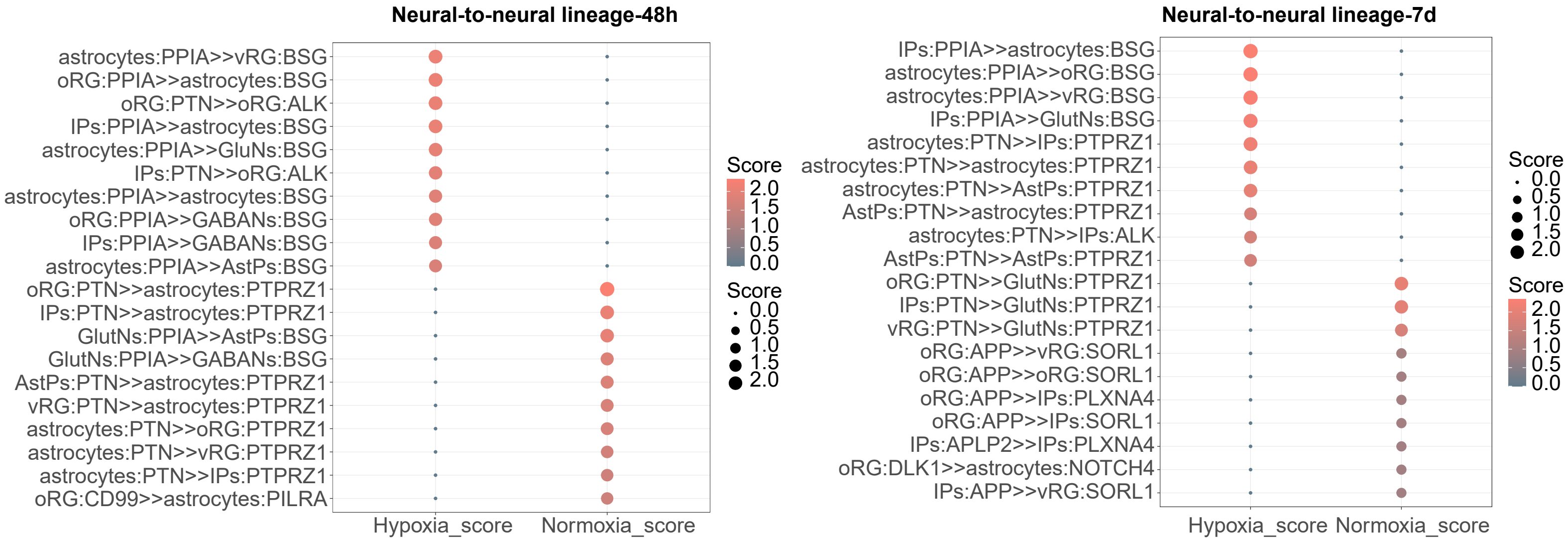

Neural-to-neural lineage-48h
astrocytes:PPIA>>vRG:BSG
oRG:PPIA>>astrocytes:BSG
oRG:PTN>>oRG:ALK
IPs:PPIA>>astrocytes:BSG
astrocytes:PPIA>>GluNs:BSG
IPs:PTN>>oRG:ALK
astrocytes:PPIA>>astrocytes:BSG
oRG:PPIA>>GABANs:BSG
IPs:PPIA>>GABANs:BSG
astrocytes:PPIA>>AstPs:BSG
oRG:PTN>>astrocytes:PTPRZ1
IPs:PTN>>astrocytes:PTPRZ1
GlutNs:PPIA>>AstPs:BSG
GlutNs:PPIA>>GABANs:BSG
AstPs:PTN>>astrocytes:PTPRZ1
vRG:PTN>>astrocytes:PTPRZ1
astrocytes:PTN>>oRG:PTPRZ1
astrocytes:PTN>>vRG:PTPRZ1
astrocytes:PTN>>IPs:PTPRZ1
oRG:CD99>>astrocytes:PILRA
Hypoxia_score
Normoxia_score
Score
2.0
1.5
1.0
0.5
0.0
Score
0.0
0.5
1.0
1.5
2.0
Neural-to-neural lineage-7d
IPs:PPIA>>astrocytes:BSG
astrocytes:PPIA>>oRG:BSG
astrocytes:PPIA>>vRG:BSG
IPs:PPIA>>GlutNs:BSG
astrocytes:PTN>>IPs:PTPRZ1
astrocytes:PTN>>astrocytes:PTPRZ1
astrocytes:PTN>>AstPs:PTPRZ1
AstPs:PTN>>astrocytes:PTPRZ1
astrocytes:PTN>>IPs:ALK
AstPs:PTN>>AstPs:PTPRZ1
oRG:PTN>>GlutNs:PTPRZ1
IPs:PTN>>GlutNs:PTPRZ1
vRG:PTN>>GlutNs:PTPRZ1
oRG:APP>>vRG:SORL1
oRG:APP>>oRG:SORL1
oRG:APP>>IPs:PLXNA4
oRG:APP>>IPs:SORL1
IPs:APLP2>>IPs:PLXNA4
oRG:DLK1>>astrocytes:NOTCH4
IPs:APP>>vRG:SORL1
Hypoxia_score
Normoxia_score
Score
0.0
0.5
1.0
1.5
2.0
Score
2.0
1.5
1.0
0.5
0.0

Figure S9

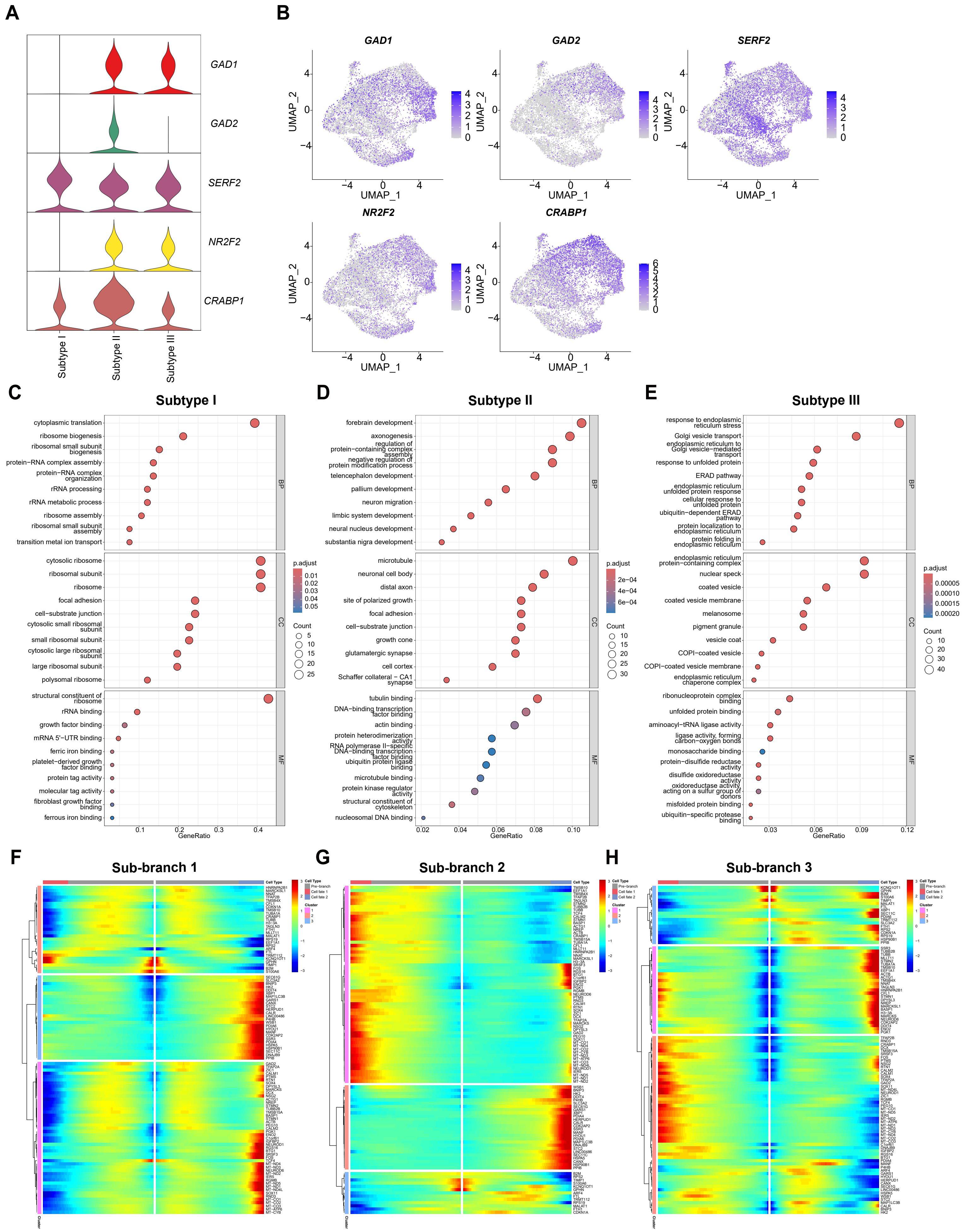

A
GAD1
GAD2
SERF2
NR2F2
CRABP1
Subtype I
Subtype II
Subtype III
B
GAD1
GAD2
SERF2
NR2F2
CRABP1
UMAP_1
UMAP_2
C
Subtype I
cytoplasmic translation
ribosome biogenesis
ribosomal small subunit biogenesis
protein−RNA complex assembly
protein−RNA complex organization
rRNA processing
rRNA metabolic process
ribosome assembly
ribosomal small subunit assembly
transition metal ion transport
cytosolic ribosome
ribosomal subunit
ribosome
focal adhesion
cell−substrate junction
cytosolic small ribosomal subunit
small ribosomal subunit
cytosolic large ribosomal subunit
large ribosomal subunit
polysomal ribosome
structural constituent of ribosome
rRNA binding
growth factor binding
mRNA 5'−UTR binding
ferric iron binding
platelet−derived growth factor binding
protein tag activity
molecular tag activity
fibroblast growth factor binding
ferrous iron binding
BP
CC
MF
GeneRatio
p.adjust
Count
D
Subtype II
forebrain development
axonogenesis
regulation of protein−containing complex assembly
negative regulation of protein modification process
telencephalon development
pallium development
neuron migration
limbic system development
neural nucleus development
substantia nigra development
microtubule
neuronal cell body
distal axon
site of polarized growth
focal adhesion
cell−substrate junction
growth cone
glutamatergic synapse
cell cortex
Schaffer collateral − CA1 synapse
tubulin binding
DNA−binding transcription factor binding
actin binding
protein heterodimerization activity
RNA polymerase II−specific DNA−binding transcription factor binding
ubiquitin protein ligase binding
microtubule binding
protein kinase regulator activity
structural constituent of cytoskeleton
nucleosomal DNA binding
GeneRatio
p.adjust
Count
E
Subtype III
response to endoplasmic reticulum stress
Golgi vesicle transport
endoplasmic reticulum to Golgi vesicle−mediated transport
response to unfolded protein
ERAD pathway
endoplasmic reticulum unfolded protein response
cellular response to unfolded protein
ubiquitin−dependent ERAD pathway
protein localization to endoplasmic reticulum
protein folding in endoplasmic reticulum
endoplasmic reticulum protein−containing complex
nuclear speck
coated vesicle
coated vesicle membrane
melanosome
pigment granule
vesicle coat
COPI−coated vesicle
COPI−coated vesicle membrane
endoplasmic reticulum chaperone complex
ribonucleoprotein complex binding
unfolded protein binding
aminoacyl−tRNA ligase activity
ligase activity, forming carbon−oxygen bonds
monosaccharide binding
protein−disulfide reductase activity
disulfide oxidoreductase activity
oxidoreductase activity, acting on a sulfur group of donors
misfolded protein binding
ubiquitin−specific protease binding
GeneRatio
p.adjust
Count
F
Sub-branch 1
G
Sub-branch 2
H
Sub-branch 3
Cell Type
Pre−branch
Cell fate 1
Cell fate 2
Cluster

Figure S10

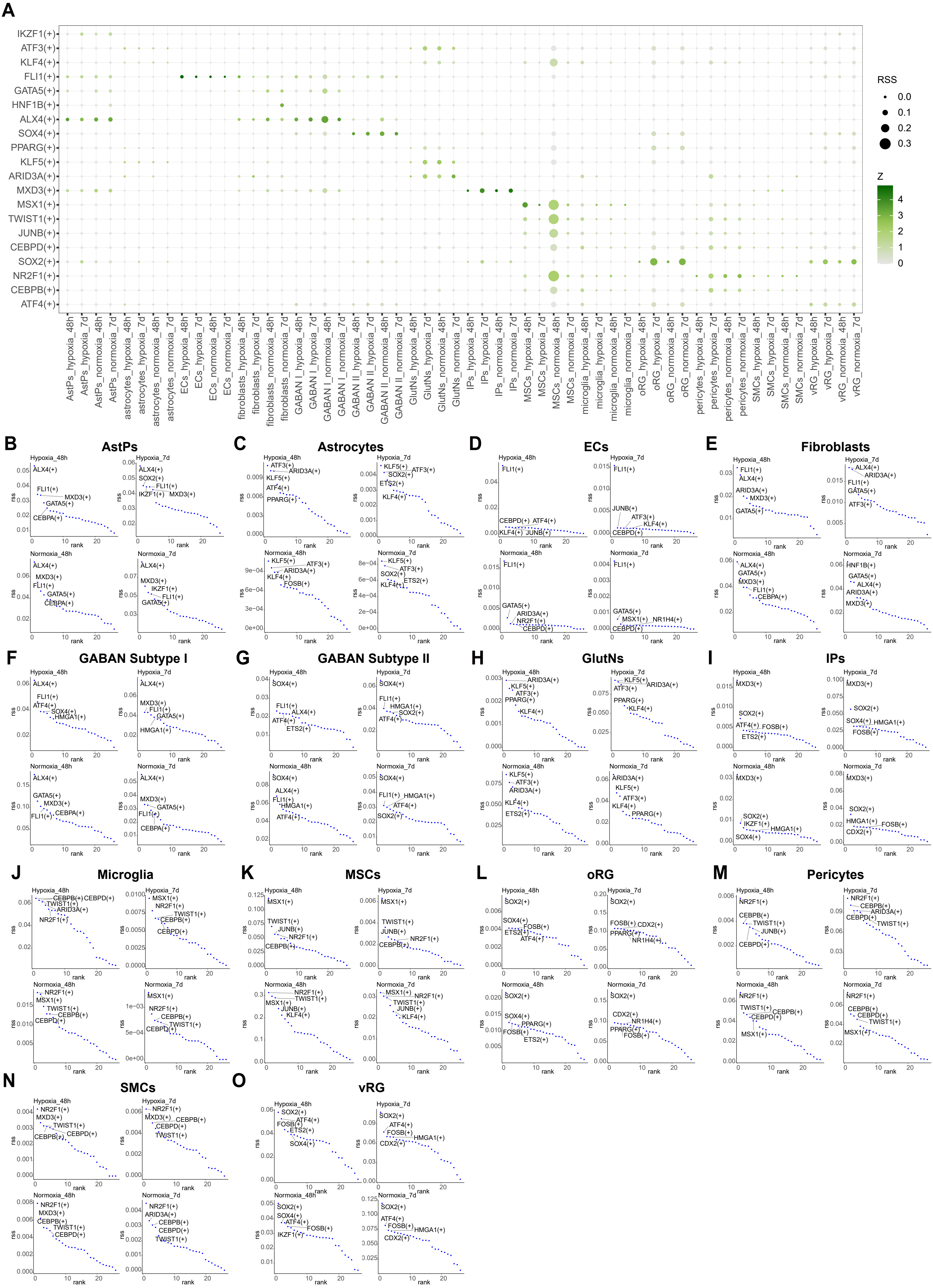
A
RSS
0.0
0.1
0.2
0.3
Z
IKZF1(+)
ATF3(+)
KLF4(+)
FLI1(+)
GATA5(+)
HNF1B(+)
ALX4(+)
SOX4(+)
PPARG(+)
KLF5(+)
ARID3A(+)
MXD3(+)
MSX1(+)
TWIST1(+)
JUNB(+)
CEBPD(+)
SOX2(+)
NR2F1(+)
CEBPB(+)
ATF4(+)
B
AstPs
C
Astrocytes
D
ECs
E
Fibroblasts
F
GABAN Subtype I
G
GABAN Subtype II
H
GlutNs
I
IPs
J
Microglia
K
MSCs
L
oRG
M
Pericytes
N
SMCs
O
vRG
Hypoxia_48h
Hypoxia_7d
Normoxia_48h
Normoxia_7d
rss
rank

Figure S11

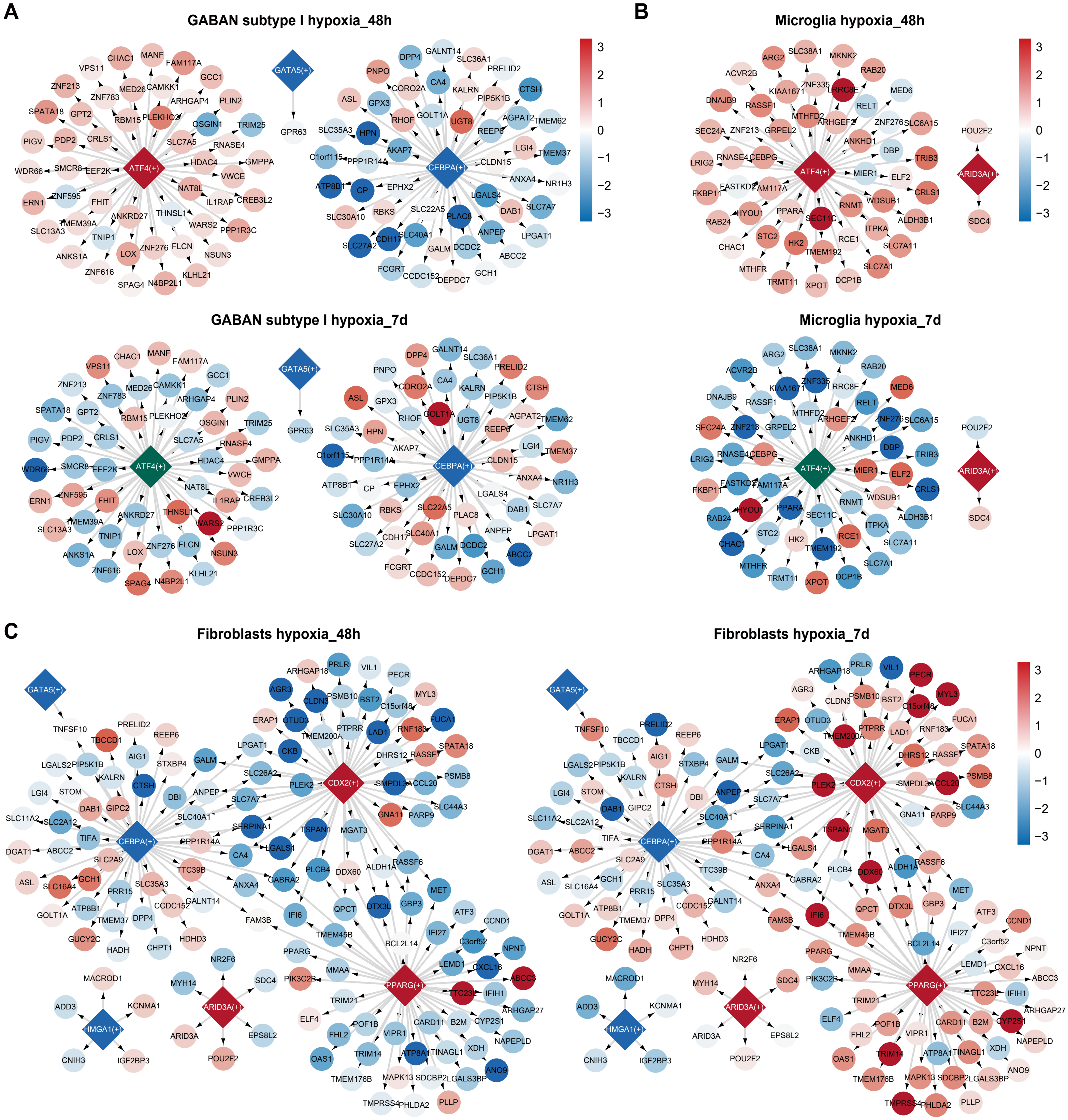

A
GABAN subtype I hypoxia_48h
GABAN subtype I hypoxia_7d
B
Microglia hypoxia_48h
Microglia hypoxia_7d
C
Fibroblasts hypoxia_48h
Fibroblasts hypoxia_7d
ATF4(+)
GATA5(+)
CEBPA(+)
ARID3A(+)
CDX2(+)
PPARG(+)
HMGA1(+)
3
2
1
0
−1
−2
−3
